# Protein Microarray Analysis of the Specificity and Cross-Reactivity of Influenza Virus Hemagglutinin-Specific Antibodies

**DOI:** 10.1128/mSphere.00592-18

**Published:** 2018-12-12

**Authors:** Rie Nakajima, Medalyn Supnet, Algis Jasinskas, Aarti Jain, Omid Taghavian, Joshua Obiero, Donald K. Milton, Wilbur H. Chen, Michael Grantham, Richard Webby, Florian Krammer, Darrick Carter, Philip L. Felgner, D. Huw Davies

**Affiliations:** a Vaccine Research and Development Center, Department of Physiology and Biophysics, University of California Irvine, Irvine, California, USA; b Maryland Institute for Applied Environmental Health, Department of Epidemiology and Biostatistics, University of Maryland, College Park, Maryland, USA; c Center for Vaccine Development and Global Health, University of Maryland School of Medicine, Baltimore, Maryland, USA; d Department of Infectious Diseases, St. Jude Children’s Research Hospital, Memphis, Tennessee, USA; e Department of Microbiology, Icahn School of Medicine at Mount Sinai, New York, New York, USA; f Infectious Disease Research Institute, Seattle, Washington, USA; University of Michigan—Ann Arbor

**Keywords:** hemagglutinin, influenza, protein microarrays

## Abstract

Current seasonal influenza virus vaccines engender antibody-mediated protection that is hemagglutinin (HA) subtype specific and relatively short-lived. Coverage for other subtypes or even variants within a subtype could be improved from a better understanding of the factors that promote HA-specific antibody cross-reactivity. Current assays to evaluate cross-reactivity, such as the ELISA, require a separate test for each antigen and are neither high-throughput nor sample-sparing. To address this need, we produced an array of 283 purified HA proteins from influenza A virus subtypes H1 to H16 and H18 and influenza B virus. To evaluate performance, arrays were probed with sera from individuals before and after a booster dose of inactivated heterologous H5N1 vaccine and naturally infected cases at presentation and follow-up during the 2010 to 2011 influenza season, when H3N2 was prevalent. The response to the H5 vaccine boost was IgG only and confined to H5 variants. The response to natural H3N2 infection consisted of IgG and IgA and was reactive with all H3 variants displayed, as well as against other group 2 HA subtypes. In both groups, responses to HA1 proteins were subtype specific. In contrast, baseline signals were higher, and responses broader, against full-length HA proteins (HA1+HA2) compared to HA1 alone. We propose that these elevated baseline signals and breadth come from the recognition of conserved epitopes in the stalk domain by cross-reactive antibodies accumulated from previous exposure(s) to seasonal influenza virus. This array is a valuable high-throughput alternative to the ELISA for monitoring specificity and cross-reactivity of HA antibodies and has many applications in vaccine development.

**IMPORTANCE** Seasonal influenza is a serious public health problem because the viral infection spreads easily from person to person and because of antigenic drift in neutralizing epitopes. Influenza vaccination is the most effective way to prevent the disease, although challenging because of the constant evolution of influenza virus subtypes. Our high-throughput protein microarrays allow for interrogation of subunit-specific IgG and IgA responses to 283 different HA proteins comprised of HA1 and HA2 domains as well as full-length HA proteins. This provides a tool that allows for novel insights into the response to exposure to influenza virus antigens. Data generated with our technology will enhance our understanding of the factors that improve the strength, breadth, and durability of vaccine-mediated immune responses and develop more effective vaccines.

## Abstract (precis)

Seasonal influenza is a serious public health problem because the viral infection spreads easily from person to person and because of antigenic drift in neutralizing epitopes. Influenza vaccination is the most effective way to prevent the disease, although challenging because of the constant evolution of influenza virus subtypes. Our high-throughput protein microarrays allow for interrogation of subunit-specific IgG and IgA responses to 283 different HA proteins comprised of HA1 and HA2 domains as well as full-length HA proteins. This provides a tool that allows for novel insights into the response to exposure to influenza virus antigens. Data generated with our technology will enhance our understanding of the factors that improve the strength, breadth, and durability of vaccine-mediated immune responses and develop more effective vaccines.

## INTRODUCTION

Despite the availability of seasonal vaccination, influenza remains a serious cause of morbidity and mortality worldwide. Seasonal influenza virus epidemics result in 290,000 to 650,000 deaths per year (1). These annual outbreaks are sustained by the amino acid substitutions in the antigenic sites of the HA1 subunit, which enable viruses to escape recognition of protective antibodies generated previously and drive antigenic drift (2–4). In addition to seasonal influenza virus outbreaks, pandemics occur at irregular intervals. The recent 2009 influenza A H1N1 pandemic resulted in >18,449 reported deaths (5, 6), although the actual number is thought to be considerably higher (7). Moreover, the potential for mortality rates in excess of a million, as seen sporadically over the past 100 years (8), remains a constant threat. Large pandemics are usually associated with the appearance of novel influenza virus subtypes in the human population. These novel subtypes originate from animal populations, and the pandemic viruses are often generated by reassortment of genetic segments between human and animal, typically avian or swine, influenza viruses (antigenic shift) (9). Highly pathogenic avian H5N1 and H7N9 viruses have caused zoonotic infections and have undergone genetic mutations and reassortment and, therefore, are considered high risk for public health. The potential for the emergence of pandemic H5N1 and H7N9 avian influenza has prompted the development of the U.S. National Prepandemic Influenza Vaccine Stockpile (10).

Influenza virus hemagglutinin (HA) proteins are divided into two phylogenetic groups: group 1 (encompassing the subtypes H1, H2, H5, H6, H8, H9, H11, H12, H13, H16, H17, and H18) and group 2 (encompassing the subtypes H3, H4, H7, H10, H14, and H15). The stalk domains of group 1 proteins share similar structures, as do the stalk domains of group 2 proteins (11). Protection against influenza virus infections is predominantly mediated by antibodies against the HA molecule on the virion surface. The HA binds to sialic acids in membrane glycoproteins and glycolipids on host cells, and antibodies are able to inhibit this interaction. Immunological and structural studies have revealed 4 or 5 important antigenic sites on the exposed head domain of the HA molecule, mutations within each of which are thought to promote antigenic drift and escape from a preexisting polyclonal response (2, 3, 12–14).

The majority of current seasonal vaccines are produced from inactivated virions of the strain(s) predicted to be prevalent in advance of the influenza season. Antigenic drift causes seasonal protection to be short-lived, requiring frequent updating of vaccine antigens and vaccine readministration. There is thus considerable interest in the development of universal influenza virus vaccines (15–17), designed to elicit cross-reactive or broadly neutralizing antibodies (bnAbs), that would reduce the need for annual vaccination and also allow a broadly protective prepandemic vaccine to be stockpiled. The presence of bnAbs in humans against influenza virus glycoproteins was not fully appreciated until the 2009 H1N1 pandemic (18), thereby raising hopes that such a vaccine is possible. bnAbs target conserved structures on the virus, notably in the HA stalk (19–21), the receptor binding pocket in the HA head (19, 22), and surface neuraminidase (NA) (23, 24).

Recent advances in producing bnAbs from individual human B cells are providing a clearer understanding of immunity to influenza virus (25) as well as other important viruses (26–28). However, the development of high-throughput tools to measure cross-reactivity of polyclonal serum lags behind, particularly to antigens that show high divergence, such as H1, H3, N1, and N2 of influenza virus. A number of protein microarrays have been produced previously to address this (29–33). However, these arrays had fewer numbers of HA proteins and coverage of fewer virus subtypes than our current high-density microarray, which comprises 283 purified HA variant proteins derived from 17 influenza A virus subtypes (H1 to H16 and H18) and influenza B virus strains. For H5, the array included both the American and the Eurasian lineages. Importantly, most H5 proteins on the array were derived from the Asian A/goose/Guangdong/1996 lineage and included diverse clade 0, 1, 2.1.3, 2.2, 2.3.2.1, 2.3.4, 2.5, 3, 4, 5, and 7 isolates. Another focus of this study was analysis of sera from H3N2-infected individuals. For a good resolution of this response, we included H3 HAs from the 1968 pandemic, modern and historic vaccine strains, and currently circulating strains, as well as several swine and equine H3 HAs. HA molecules were expressed as HA1 only or as full-length HA1+HA2 molecules. These arrays were probed with available sera from naturally infected patients with laboratory-confirmed influenza virus infection during the 2010 to 2011 influenza season or from individuals boosted with an H5N1 vaccine. Distinct reactivity patterns were detected after H3N2 natural infection and after H5N1 booster vaccination. The data presented here demonstrate the potential of this assay to aid epidemiological surveys, guide vaccine development, and extend our understanding of the antibody response against influenza viruses.

## RESULTS

### The antibody response against HA1 proteins is subtype specific after boosting with an H5N1 vaccine and during H3N2 natural exposure.

**FIG 1:**
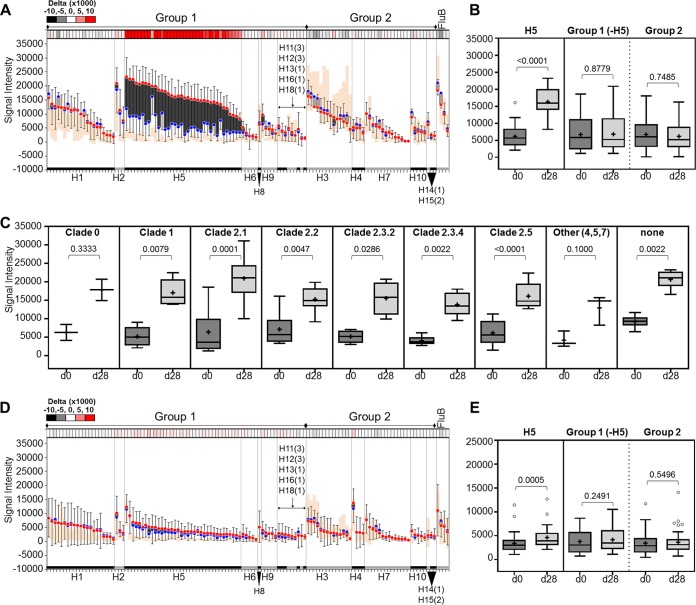
Antibody reactivity against HA1 proteins after vaccination. Anti-IgG (A, B, and C) and anti-IgA (D and E) antibody responses after boosting with H5 vaccine are depicted as floating bar graphs or as Tukey box plots. Bar graphs are sorted by subtype and decreasing signal intensity (SI) at d28. Each bar represents the difference between the average signal intensities of two time points for a strain (blue dots, d0 SI; red dots, d28 SI; error bars, standard deviations [SD], *n* = 10). Dark bars indicate a positive difference between the two time points (d28 > d0), while light bars indicate a negative difference (d0 > d28). A heat map of delta values (d28 − d0) is shown above each graph. The orange area represents the signal intensity distribution (average ± 1 SD, *n* = 11) of a reference group (GCRC) with no active influenza virus infection or history of H5 vaccination. The Tukey box plots in panels B and E show H5 alone and pooled group 1 or group 2 subtype signal intensities. Panel C shows average signal intensities at the two time points among the various H5 clades. Means are indicated by “+.” The two-tailed Mann-Whitney test for unpaired data was used to calculate statistical significance between the two time points, where *P* < 0.05 defines statistical significance. HA1, hemagglutinin head domain; d0, day 0; d28, day 28; GCRC, General Clinical Research Center (UC Irvine).

**FIG 2:**
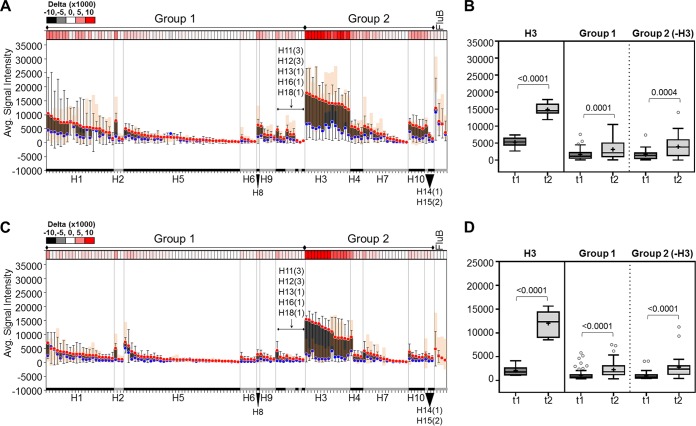
Antibody reactivity against HA1 proteins during natural exposure. Anti-IgG (A and B) and anti-IgA (C and D) antibody responses during H3-confirmed natural exposure are depicted as floating bar graphs or as Tukey box plots. Bar graphs are sorted by subtype and decreasing signal intensity (SI) at t2. Each bar represents the difference between the average signal intensities of two time points for a strain (blue dots, t1 SI; red dots, t2 SI; error bars = SD, *n* = 5). Dark bars indicate a positive difference between the two time points (t2 > t1), while light bars indicate a negative difference (t1 > t2). A heat map of delta values (t2 − t1) is shown above each graph. The orange area represents the signal intensity distribution (average ± 1 SD, *n* = 11) of a reference group (GCRC) with no active influenza virus infection or history of H5 vaccination. The Tukey box plots show H3 alone and pooled group 1 or group 2 subtype signal intensities. Means are indicated by “+.” The two-tailed Mann-Whitney test for unpaired data was used to calculate statistical significance between the two time points, where *P* < 0.05 defines statistical significance. HA1, hemagglutinin head domain; t1, time point 1; t2, time point 2; GCRC, General Clinical Research Center (UC Irvine).

**FIG 3:**
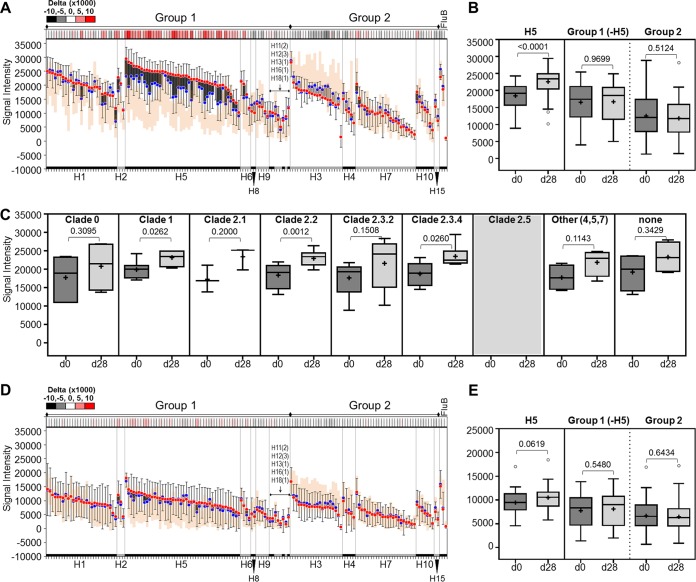
Antibody reactivity against whole HA (HA1+HA2) proteins after vaccination. Anti-IgG (A, B, and C) and anti-IgA (D and E) antibody responses after boosting with H5 vaccine are depicted as floating bar graphs or as Tukey box plots. Bar graphs are sorted by subtype and decreasing signal intensity (SI) at d28. Each bar represents the difference between the average signal intensities of two time points for a strain (blue dots, d0 SI; red dots, d28 SI; error bars, SD, *n* = 10). Dark bars indicate a positive difference between the two time points (d28 > d0), while light bars indicate a negative difference (d0 > d28). A heat map of delta values (d28 − d0) is shown above each graph. The orange area represents the signal intensity distribution (average ± 1 SD, *n* = 11) of a reference group (GCRC) with no active influenza virus infection or history of H5 vaccination. The Tukey box plots in panels B and E show H5 alone and pooled group 1 or group 2 subtype signal intensities. Panel C shows average signal intensities at the two time points among the various H5 clades; clade 2.5 is not represented in this data set. Means are indicated by “+.” The two-tailed Mann-Whitney test for unpaired data was used to calculate statistical significance between the two time points, where *P* < 0.05 defines statistical significance. HA1, hemagglutinin head domain; d0, day 0; d28, day 28; GCRC, General Clinical Research Center (UC Irvine).

**FIG 4:**
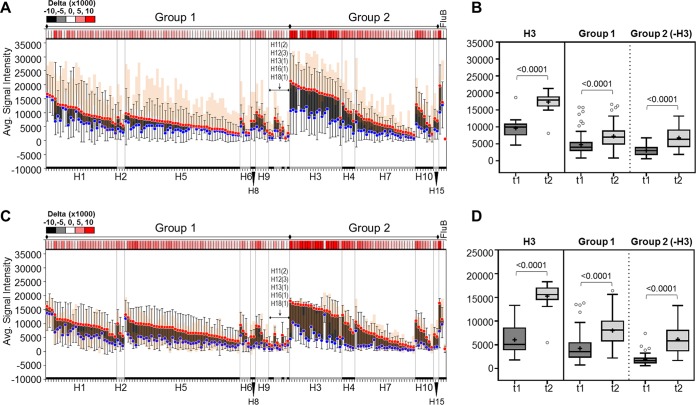
Antibody reactivity against whole HA (HA1+HA2) proteins during natural exposure. Anti-IgG (A and B) and anti-IgA (C and D) antibody responses during H3-confirmed natural exposure are depicted as floating bar graphs or as Tukey box plots. Bar graphs are sorted by subtype and decreasing signal intensity (SI) at t2. Each bar represents the difference between the average signal intensities of two time points for a strain (blue dots, t1 SI; red dots, t2 SI; error bars, SD, *n* = 5). Dark bars indicate a positive difference between the two time points (t2 > t1), while light bars indicate a negative difference (t1 > t2). A heat map of delta values (t2 − t1) is shown above each graph. The orange area represents the signal intensity distribution (average ± 1 SD, *n* = 11) of a reference group (GCRC) with no active influenza virus infection or history of H5 vaccination. The Tukey box plots show H3 alone and pooled group 1 or group 2 subtype signal intensities. Means are indicated by “+.” The two-tailed Mann-Whitney test for unpaired data was used to calculate statistical significance between the two time points, where *P* < 0.05 defines statistical significance. HA1, hemagglutinin head domain; t1, time point 1; t2, time point 2; GCRC, General Clinical Research Center (UC Irvine).

**(i) Boosting with an H5N1 vaccine generates a robust subtype-specific IgG but no IgA response to HA1 proteins.** In a previous clinical trial (clinicaltrials.gov ID NCT00680069), the safety and efficacy of administering different clades of H5N1 at prime and at boost were investigated (34). In a substudy of that trial, subjects were primed twice with either low-dosage or high-dosage nonadjuvanted H5N1 vaccine derived from A/Vietnam/1203/2004 (clade 1) and were boosted with a single intramuscular high dosage of a different H5N1 vaccine derived from the antigenically distinct A/Indonesia/05/05 (clade 2) virus more than 1 year later (see Fig. S1 in the supplemental material). The serum samples used in our microarray study were obtained from 25 subjects, 10 of whom were given the high-dosage primary series, and the remaining 15 the low-dosage primary series. All 25 subjects were eventually boosted with a high-dose boost. Data presented in Fig. 1 to 4 were obtained from the high-dosage primary series group only.

10.1128/mSphere.00592-18.1 FIG S1 Schematic of sample collection for protein microarray probing: (A) Natural infection; (B) H5N1 vaccine trial (https://clinicaltrials.gov/show/NCT00680069). Serum samples probed on protein microarrays are indicated by blood drops. Download FIG S1, TIF file, 0.4 MB. Copyright © 2018 Nakajima et al. 2018 Nakajima et al. This content is distributed under the terms of the Creative Commons Attribution 4.0 International license.

Sera (*n* = 10) from individuals who received the high-dosage primary series were first interrogated here for IgG and IgA responses at two time points—preboost (d0) and 28 days postboost (d28)—using the microarray of hemagglutinin proteins expressed as HA1 or HA1+HA2 molecules. Figure 1 shows data using HA1 proteins for antibody detection in the vaccine study. H5-boosted individuals exhibit a strong IgG response to nearly all H5 variants on the HA1 panel in addition to the administered strains (Fig. 1A). This response is subtype specific as demonstrated by a nearly 3-fold increase in average signal intensity on d28 compared to d0 in the H5 subtype (*P* < 0.0001) but not in other subtypes (Fig. 1A and B; Fig. S2A). Analysis of the H5 response by clade (Fig. 1C) revealed broad reactivity across all the clades. This breadth is likely a consequence of the clade 1 prime followed by a clade 2 boost (35, 36). In addition, there seems to be a durable IgG response to H5 at least 1 year after the primary vaccination series, as illustrated by the higher preboost H5 signal intensities in vaccinees (blue circles) than in those seen in individuals with no known history of H5 vaccination (orange area). This difference is not apparent in other subtypes.

10.1128/mSphere.00592-18.2 FIG S2 Delta change of H3- or H5-specific antibody signal intensities (SI) compared to pooled group 1 or 2 hemagglutinin (HA). (A and B) IgG (A) and IgA (B) antibody responses against H5 head only or whole hemagglutinin proteins (left of dotted vertical dividers) are compared to pooled group 1 or group 2 HA responses from H5-vaccinee sera. Group 1 data pool excludes H5 subtypes. (C and D) IgG (C) and IgA (D) antibody responses against H3 head only or whole hemagglutinin proteins (left of dotted vertical divider) are compared to pooled group 1 or group 2 HA responses from H3-confirmed infection sera. Group 2 data pool excludes H3 subtypes. Delta signal intensities (d28 − d0 or t2 − t1) are depicted as Tukey box plots, with means represented by “+.” The Kruskal-Wallis test with Dunn’s multiple-comparison test was used to calculate statistical significance, where *P* < 0.05 defines statistical significance. HA1, hemagglutinin head domain; HA2, hemagglutinin stalk domain; HA1+HA2, full-length hemagglutinin; Grp, group. Download FIG S2, TIF file, 1.5 MB. Copyright © 2018 Nakajima et al. 2018 Nakajima et al. This content is distributed under the terms of the Creative Commons Attribution 4.0 International license.

In contrast to the IgG response, the overall serum IgA response to the HA1 panel is low at both time points (Fig. 1D). While the IgA response to H5 subtypes between the two time points is statistically significant (Fig. 1E; *P* = 0.0005), the magnitude of the difference is small (Fig. S2B). This is entirely consistent with an intramuscular route of entry for the vaccine compared to a mucosal route of entry seen in natural influenza virus exposure. Interestingly, the average d0 IgA response in vaccinees (blue circles) is higher than that seen in the reference group (orange area), which might suggest long-lived serum IgA against H5 HA1 proteins that persist at least 1 year after a two-prime H5N1 vaccination series. A larger study is needed to confirm these observed baseline differences.

**(ii) Natural influenza virus (H3) infection generates subtype-specific and cross-reactive IgA and IgG antibody responses to HA1 proteins.** To assess the antibody response induced against the hemagglutinin panel during active influenza virus infection, serum samples from a small group (*n* = 5) of symptomatic patients were probed on the array. Sera from individuals experiencing an active influenza virus infection during the 2010 to 2011 season were collected at time of presentation (t1 = acute infection) and at follow-up (t2 = convalescent infection), 7 to 31 days later. Samples were later confirmed to be H3 subtype positive, consistent with the dominant subtype in circulation at the time of sampling (37).

Serum samples from infected patients display the greatest increase in both IgG and IgA responses to HA1 proteins of the H3 subtype compared to other subtypes (Fig. 2). It is noteworthy that all H3 strains on the HA1 panel are reactive, indicating there is cross-reactivity within a given subtype. This could be due to the redundancy of epitope coverage provided in the polyclonal anti-HA1 response and/or the extent of epitope conservation within an HA subtype. Interestingly, there appear to be some H3 strains that react more strongly, on average, to healthy reference serum IgG (orange area), but weakly to acute-phase serum IgG (t1, blue circles) (Fig. 2A). The reference group, though healthy, is expected to have IgG antibodies to H3 proteins due to the very high likelihood that these individuals had past infections with H3; H3N2 is a commonly encountered circulating strain and a component of seasonal vaccines. In addition, there seem to be some cross-reactive IgG responses to other subtypes, including H1, H9, and H10, which are not observed in the vaccine group (Fig. 1A). Though statistically significant, the magnitude of the seroconversion among these non-H3 subtypes, indicated by the length of the bars, is far lower than that seen in H3 strains (Fig. 2B; Fig. S2C).

The robust subtype-specific IgA response to the HA1 panel seen in this cohort (Fig. 2C and D; Fig. S2D) contrasts with the very low IgA response observed in the vaccine group. This is consistent with mucosal exposure, which is the presumed route through which these patients were infected. The average patient IgA response to some H3 strains, curiously, is higher at convalescence (t2, red circles) than seen in the healthy reference group (orange area) (Fig. 2C). A study consisting of a much larger cohort of patients with acute- and convalescent-phase influenza virus infections may confirm these preliminary findings.

### The antibody response against whole HA proteins (HA1+HA2) is subtype specific after boosting with an H5N1 vaccine but appears cross-reactive in H3N2 natural exposure.

**(i) Boosting with an H5N1 vaccine generates a subtype-specific IgG response to HA1+HA2 proteins.** The IgG and IgA response profiles of serum samples from H5-boosted vaccinees to the HA1+HA2 panel are similar to those found against the HA1 panel, where a subtype-specific IgG response is detected, but not IgA (Fig. 3; Fig. S2A and B). The magnitude of seroconversion to IgG by d28, indicated by the length of the bars, however, is lower than that seen with HA1 (Fig. 3A and B; Fig. S2A). Similar to the reactivity against the HA1 panel, the average baseline IgG response to H5 subtypes on the HA1+HA2 panel (blue circles) is higher than the average response of the reference group (orange area) (Fig. 3A). This may indicate long-lived anti-H5 antibodies induced by the priming vaccination. Of note, unlike signals against the HA1 panel, IgG signals against HA1+HA2 from all subtypes were already elevated at the first time point (Fig. 3B versus Fig. 1B). This was also observed to a lesser degree in the infected group. Overall, this is suggestive of improved detection of preexisting antibody by the HA1+HA2 antigen, presumably by detection of antibodies against HA2, which forms part of the conserved stalk region (see Discussion).

Similar to findings using HA1 for detection, the H5 response to HA1+HA2 revealed broad reactivity across the different clades (Fig. 3C), again presumably because of the clade 1 followed by clade 2 vaccination regimen. Although IgA signals against the H5 subtype variants are significantly elevated at 28 days postboost (Fig. 3D and E), the magnitude of this increase is less than that seen for IgG. This observation that the vaccine boosts H5 IgG responses more than IgA against the whole molecule is consistent with similar differential isotype response against the HA1 panel (Fig. 1).

**(ii) Natural H3N2 influenza virus infection generates subtype-specific and cross-reactive IgA and IgG antibody responses to HA1+HA2 proteins.** Patients naturally exposed to influenza show H3-specific IgG and IgA responses to the HA1+HA2 panel (Fig. 4; Fig. S2C and D). Like those observed in H5 vaccinees, these responses are elevated at the first time point (t1, blue dots), again presumably because of detection of antibodies to HA2. Remarkably, unlike the H3-specific response seen when using HA1 alone, the response to HA1+HA2 was broadly reactive across other group 2 subtypes, as well as group 1 subtypes and influenza B virus, as demonstrated by the increase in signal intensity upon follow-up (t2, red dots). This is seen in both IgG (Fig. 4A and B) and IgA (Fig. 4C and D). On average, IgG signal intensities of the reference group against this panel are higher than those seen in these patients even at follow-up (Fig. 4A, red dots versus orange area). In contrast, the IgA profiles of the two groups look similar. The reasons for the differences seen between reference and patient group profiles are unclear, though they may be attributable to an individual’s age or health.

### Recipients of an H5 vaccine booster dose, after either low- or high-dosage primary series H5 vaccine, show significantly higher specific IgG responses to H5 molecules.

**FIG 5:**
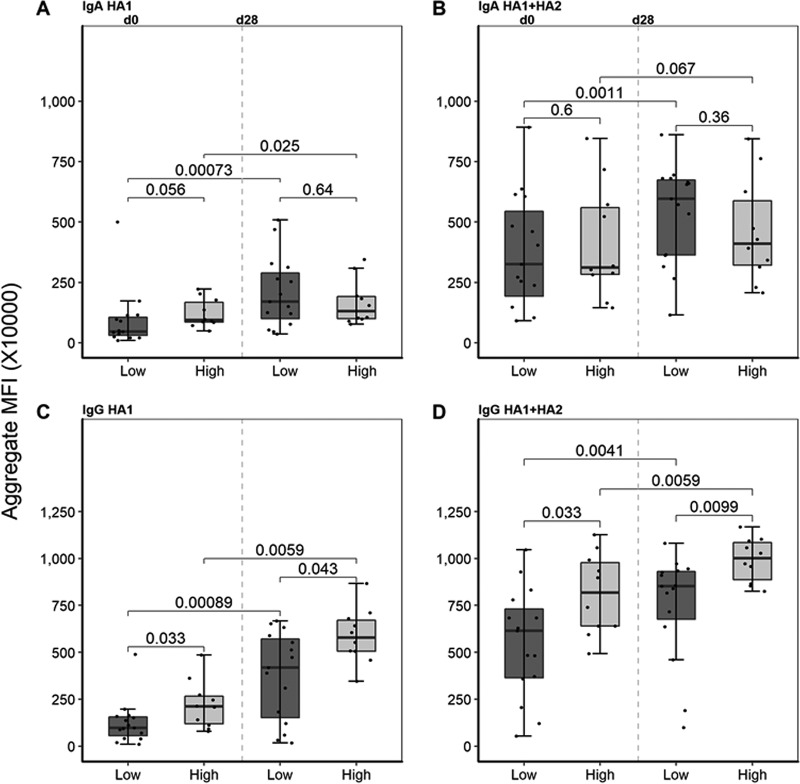
IgA and IgG aggregated signal intensities in low-dose and high-dose H5 vaccine recipients. H5 vaccine recipients are stratified by the vaccine dose that they received 1 year prior to boost (low, 15 µg; high, 90 µg). Their antibody reactivities against H5 strains at two time points, d0 and d28, are depicted as Tukey box plots, with medians represented by horizontal bars. “d0” refers to 1 year after primary vaccination, and “d28” refers to 28 days after receiving a 90-µg boost. (A and B) IgA antibody responses of low- and high-dose vaccinees, against HA1 only (A) and HA1+HA2 proteins (B) on d0 and d28. (C and D) IgG antibody responses of low- and high-dose vaccinees, against HA1 only (C) and HA1+HA2 proteins (D) on d0 and d28. Differences between high- and low-dose groups were analyzed using the Wilcoxon rank sum test, and those between time points were analyzed using the Wilcoxon signed-rank test where significance was set at *P* < 0.05.

The paired serum samples analyzed in this substudy were derived from subjects who received two doses of either a high (90 µg) or a low (15 µg) dosage of the H5N1 A/Vietnam/1203/2004 (clade 1) influenza virus vaccine as the primary series. Subsequently, all subjects received a single high-dosage (90 µg) H5N1 A/Indonesia/05/05 (clade 2) vaccine. The results in Fig. 5A and B show no significant difference in the IgA levels between individuals who received either the high- or low-dosage priming at baseline (d0) or at 28 days postboost (d28). The day 0 time point gives the relative antibody remaining greater than 1 year after the high- or low-dosage vaccine. In contrast, subjects who received high- or low-priming-dosage vaccine show a statistically significant difference in the baseline (d0) IgG antibody response to HA1 (*P* = 0.033) and HA1+HA2 (*P* = 0.033) (Fig. 5C and D). Similarly, the level of IgG is significantly higher after boost (d28) for subjects who received the high-dosage priming. The magnitude of increase in antibody response between d0 and d28 for HA1 and HA1+HA2 after boosting is not significantly affected by the vaccine dosage from the primary series (Fig. S3). These data indicate that the most efficient anamnestic responses were not necessarily associated with the higher-dosage heterologous primary series vaccination.

10.1128/mSphere.00592-18.3 FIG S3 Delta change (d28 − d0) of aggregated signal intensities for H5 antigens. H5 vaccine recipients are stratified by the vaccine dose that they received one year prior to boost (low, 15 µg; high, 90 µg). Their antibody reactivity changes against H5 strains at two time points, d0 and d28, are depicted as Tukey box plots, with medians represented by horizontal bars. (A and B) IgA antibody response change of low- and high-dose vaccinees between d0 and d28, against HA1 only (A) and HA1+HA2 proteins (B). (C and D) IgG antibody responses of low- and high-dose vaccinees between d0 and d28, against HA1 only (C) and HA1+HA2 proteins (D). A two-tailed Wilcoxon rank sum test for unpaired data was used to calculate statistical significance between dose groups, where *P* < 0.05 defines statistical significance. Download FIG S3, TIF file, 0.6 MB. Copyright © 2018 Nakajima et al. 2018 Nakajima et al. This content is distributed under the terms of the Creative Commons Attribution 4.0 International license.

## DISCUSSION

In the U.S., there are no killed whole-virion influenza virus vaccines. FluMist (Medimmune Vaccines, approved since 2003) is the only currently available live, attenuated whole-virion vaccine. Subunit vaccines in the U.S. are recombinantly expressed HA vaccine (Flublok, Protein Sciences, approved since 2013). All other vaccines are technically split vaccines (i.e., whole virus disrupted by detergents and then purified to a desired HA content). Recombinant influenza virus vaccines would be beneficial in the event of a pandemic or a shortage of vaccine supply because of the shorter processing time required for large-scale manufacture and independence from an egg supply. Moreover, virus propagation often results in HA mutations adapted for growth in embryonated chicken eggs, which can affect the antigenicity of the virus (38–40).

Determining which specific virus of a given hemagglutinin (HA) subtype is included in seasonal influenza virus vaccines is guided by predictions of the viruses that will dominate in a future outbreak. The strategy is suboptimal considering that the immunity engendered by current vaccination approaches is generally subtype specific or even strain specific, and efficacy against strains that emerge in subsequent epidemics and pandemics is unpredictable. The rules for predicting and enhancing strain coverage engendered by seasonal influenza virus vaccination are not well understood but would benefit from a clearer understanding of the extent of HA cross-reactivity engendered by vaccination and infection.

To develop a high-throughput way to measure cross-reactivity of polyclonal serum, particularly to antigens that show high divergence, we evaluated a high-density microarray that consisted of 283 purified HA variant proteins derived from 17 influenza A virus subtypes (H1 to H16 and H18), with HA molecules expressed as HA1 only or as HA1+HA2 full-length molecules. Using this large number of proteins, compared to smaller numbers used in previous studies (30–33, 41), allowed for the inclusion of both HA1 and full-length HA proteins (in contrast to some earlier studies that used only HA1 [30–32]). HA1 is more likely to detect strain-specific responses, while full-length HA contains the conserved stalk domain and will also detect cross-reactive antibodies that target this region. In addition, in some settings a response to an influenza virus vaccine can be very strain specific without boosting cross-reactive antibodies. In this case, the inclusion of a large variety of antigenically different HAs increases the chances of detecting this response.

Here, we first examined antibody profiles from an H5N1 vaccine study and demonstrated that a heterologous (clade 1-clade 2) prime-boost vaccination gave a broadly cross-reactive H5 IgG antibody response across all the clades represented on the array. This is consistent with a report using samples from the same clinical trial (34) in which higher hemagglutination inhibition and microneutralization geometric mean titers were seen to both clade 1 and 2 strains in subjects primed with clade 1 vaccine and later boosted with clade 2 vaccine, compared to naive subjects who received two doses of clade 2 vaccine 28 days apart. In another study, broad levels of H5 cross-reactivity were also achieved in humans using recombinant vaccinia virus Ankara (MVA) to deliver a clade 1 H5 (42). Cross-reactivity was induced against both clade 1 and clade 2 H5 variants, although the titers were overall higher for the homologous, clade 1 H5s. It has also been reported that the priming dosage of a clade 1 H5 vaccine does not differentially impact the IgG response to a subsequent dose of the clade 2 H5-specific vaccine (43). However, we observed significantly higher IgG signals at the higher dose (90 µg versus 15 µg), not only 1 year after the priming regimen but also after the clade 2 booster immunization. These data would support the notion that a higher dose for priming might be preferable.

It has been described that vaccination with one dose of an H5N1 vaccine boosted cross-reactive antibody responses to the stalk domain while a booster dose of H5N1 vaccine induces mostly antibodies that target the head domain of HA (44, 45). We found that booster vaccination with the clade 2 H5 vaccine induced antibodies reactive with other group 1 whole HA (HA1+HA2) inefficiently. This is expected since these individuals had already been primed with an H5 vaccine, and the booster dose, therefore, likely induced a recall response that was specific to the head domain of the H5. In addition, we studied subjects naturally infected with H3N2 influenza virus sampled during the 2010 to 2011 influenza season. Natural infection engendered a broadly cross-reactive response within the subtype experienced during infection, in this case H3. Highlighting this was the finding that natural H3N2 infection boosted IgA responses against all H3 variants displayed. Encouragingly, we also found that H3N2 infection boosted antibody responses to group 2 HAs. These finding are similar to a recent study that analyzed sera from H3N2-infected individuals for cross-reactivity by ELISA—a highly sensitive but low-throughput assay format (46).

We also observed that H3N2 infection elicited both IgA and IgG. IgA, particularly secretory IgA (S-IgA), is likely to be the primary means of protection acquired against influenza virus that infects via the respiratory mucosa, although other Ig isotypes and cellular mechanisms can also play a protective role in the absence of IgA (47–51). Live attenuated influenza virus vaccine (LAIV) is administered intranasally and induces both S-IgA and IgG in the upper respiratory tract, in part by mimicking the natural route of entry. LAIV also induces broader immunity against antigenically drifted strains than inactivated vaccines (52). The efficacy of inactivated seasonal vaccines against influenza may be improved if the induction of IgA could be enhanced, such as by intranasal delivery (53, 54).

Interestingly, we consistently observed preexisting heterosubtypic antibody at day 0 in both seasonal cases and vaccinees when using full-length HA1+HA2 as detection antigen, compared to HA1 alone. This elevated baseline has the effect of reducing the fold increase at the second time point, thus making the effect of the clade 2 boosting appear more dramatic when using the HA1 alone. One explanation is that the conformation of HA1 is more authentic when assembled in the full-length molecule and therefore better suited for detection of preexisting antibody. Alternatively, and more likely, the preexisting antibodies are recognizing epitopes in the full-length molecule not present in HA1 alone, i.e., the HA2 polypeptide that, with part of HA1, forms the stalk domain. This is consistent with the notion that repeated influenza virus exposure(s) and/or vaccinations to different variants result each time in a primary, strain-specific response to the variable globular head domain but boosting and gradual accumulation of antibodies against the conserved stalk region (46, 55).

Although the array can help characterize specificity and cross-reactivity, additional functional assays such as virus neutralization or ADCC assays are required to assess the immunological significance of the antibodies detected. Although the majority of neutralizing epitopes in HA map to the globular head, binding of antibodies to HA in the microarray may not necessarily correlate with protection. Moreover, head-reactive neutralizing antibodies typically show limited cross-reactivity. Indeed, cross-reactivity may correlate better with ADCC as these antibodies preferentially recognize epitopes of the stalk region of HA and are located mainly in HA2. Of note, recent studies have provided evidence that nonneutralizing, broadly binding antibodies (isolated from both mice and humans) can provide strong protection, at least in animal models (56–58).

It is also important to discuss the limitations of this technology. The data generated using the array are only as good as the recombinant HA probes used. Misfolded or denatured HA will detect different antibodies than correctly folded HA. The system used to express the antigens might also play a role since, e.g., bacterial systems typically do not attach glycans while glycan sizes vary between insect cell- and different mammalian cell-based expression systems. Differences in glycosylation might influence the detected antibody response as well. Also, while large amounts of data can be generated via influenza virus protein arrays, excellent quality control needs to be in place to account for batch-to-batch variations, trending, printing errors, and other technical issues. Finally, as mentioned above, only binding antibody can be measured, and functionality needs to be assessed using additional assays.

Nevertheless, this study supports the utility of the influenza virus purified HA protein microarray as a rapid and high-throughput tool to survey seroreactivity against hundreds of HA variants and inform vaccine and adjuvant development. Here, we investigated antibody profiles induced by H5N1 vaccination and H3N2 natural infection. While we observed interesting differences, these two types of exposures cannot be directly compared since humans are naive to H5N1 but not to H3N2 and since the HAs of the two viruses belong to different HA groups. In future studies, we will therefore investigate differences between exposure to the same virus subtype via natural infection and via vaccines administered by different routes, different dosing regimens, adjuvants, and excipients, to advance our understanding of factors that improve the strength, breadth, and durability of vaccine-mediated immune responses and discover and develop more effective vaccines.

## MATERIALS AND METHODS

Methods were performed in accordance with relevant regulations and guidelines.

### Human sera.

**(i) Naturally infected sera.** Sera from five patients with RT-PCR-confirmed (59) influenza virus infection (H3 subtype positive) were collected during the 2010 to 2011 season in Memphis, TN, USA. This study was conducted with informed consent and approved by the Institutional Review Board (IRB protocol number XPD09-078) of St. Jude Children’s Research Hospital, Memphis, TN. Sera were provided to the University of California Irvine (UCI) for assay without patient identifiers and were classified as exempt by the UCI IRB. Sera were collected at two time points: at time of presentation (“t1” = acute-phase infection), and at follow-up (“t2” = convalescent-phase infection) 7 to 31 days later.

**(ii) H5N1 vaccine study.** Sera from 25 subjects (age 18 to 64 years) were obtained from future-use consented specimens archived from a clinical trial during which a single intramuscular low-dosage (15 µg) or high-dosage (90 µg) booster dose of an inactivated subvirion A/Indonesia/05/05 (clade 2) H5N1 vaccine was administered more than 1 year (1.4 to 3.7 years) after receipt of various dosages of a two-dose primary series with a clade 1 (A/Vietnam/1203/2004) H5N1 vaccine (identifier NCT00680069) (34) (see Fig. S1 in the supplemental material). Samples were collected from boosted individuals at baseline (d0) and 28 days after the boost (d28) and used to probe protein arrays. All 25 paired serum specimens were from individuals who received the high-dosage (90 µg) clade 2 booster vaccine; 10 paired serum specimens were from high-dosage (30 to 90 µg) clade 1 primary series recipients and 15 paired serum specimens were from low-dosage (3.75 to 15 µg) clade 1 primary series recipients. Sera were provided to the University of California Irvine (UCI) for assay without patient identifiers and were classified as exempt by the UCI IRB and University of Maryland, Baltimore (UMB) IRB.

**(iii) Control sera.** As a reference, serum samples collected in early 2008 from healthy blood donors at UC Irvine’s General Clinical Research Center (GCRC) who were not known to have received H5N1 vaccine were probed on the arrays. Throughout that season, H1N1, H3N2, and influenza B viruses cocirculated, with H3N2 being the most commonly reported strain (60).

### Protein microarray manufacture and probing.

**(i) Protein microarrays.** HA protein microarrays were fabricated as previously described (61) with modifications. Briefly, purified influenza virus antigens, representing 17 influenza A virus subtypes and influenza B virus, were obtained from Sino Biological Inc. (Beijing, China) (Fig. S4; Table S1). The protein set comprised 283 lyophilized influenza virus hemagglutinin (HA), 4 neuraminidase (NA), and 1 nucleoprotein (NP) variants, with HA molecules expressed either as separate subunits (HA1 or HA2) or as a whole molecule (HA1+HA2). The neuraminidase, nucleoprotein, HA2, and NS1 features were not used for the analysis in this study. Proteins were derived from roughly equal numbers of human and nonhuman (avian, swine, etc.) isolates and were expressed in baculovirus or human cell expression systems. Approximately half of the total HA1+HA2 molecules printed on the array were expressed in a baculovirus system, and 2% of total HA1 molecules were similarly expressed (Fig. S4C). H5 strains are highly represented on the array, making up almost a third of the HA protein set, and included HAs from the American and Eurasian lineage. The majority of the Eurasian lineage HAs were derived from the A/goose/Guangdong/1996 virus, which gave rise to the highly pathogenic Asian H5N1 viruses. HAs from clades 0, 1, 2.1.3, 2.2, 2.3.2.1, 2.3.4, 2.5, 3, 4, 5, and 7 were included. This was followed by H1 (17%), H3 (16%, including H3 HAs from the 1968 pandemic, modern and historic vaccine strains, and currently circulating strains as well as several swine and equine H3 virus HAs), and H7 (12%) subtypes, which are relevant for being commonly encountered during seasonal epidemics (Fig. S4A). Proteins expressed in insect cells presented signal intensities that were overall higher than corresponding ones expressed in human cells as demonstrated by the low transformed human-cell-to-baculovirus ratios (Fig. S5). The same conclusions were drawn whether the whole data set was pooled or whether data from the two expression systems were analyzed independently in this pilot study. Therefore, analyses from the pooled data set are presented throughout. Prior to printing, each lyophilized antigen was reconstituted to a concentration of 0.1 mg/ml in phosphate-buffered saline (PBS) with 0.001% Tween 20 (T-PBS). In addition to these purified proteins, 14 nonstructural (NS1) antigens were expressed by an Escherichia coli-based *in vitro* transcription/translation (IVTT) system (RTS 100 E. coli HY kit; Biotechrabbit GmbH, Hennigsdorf, Germany) from purified DNA. Negative control (“No DNA” control) was included by performing IVTT reactions in the absence of DNA template. Purified proteins and crude IVTT reaction mixtures were printed onto nitrocellulose-coated glass AVID slides (Grace Bio-Labs, Inc., Bend, OR, USA) using an Omni Grid 100 microarray printer (Genomic Solutions).

10.1128/mSphere.00592-18.4 FIG S4 Influenza microarray. (A) Seventeen influenza A virus subtypes (H1 to H16 and H18) and four influenza B virus strains were represented on the arrays for a total of 153 unique strains. (B) The influenza virus microarray comprised a total of 302 hemagglutinin (HA), neuraminidase (NA), nucleoprotein (NP), and nonstructural (NS) proteins derived from roughly equal human and nonhuman isolates. Two proteins were either biotinylated or human Fc tagged and so were removed from further analysis. (C) Proteins were expressed from three systems: human cells, baculovirus, or cell free (*in vitro* transcription and translation [IVTT]). Only NS proteins were expressed by IVTT. The bar graph depicts the total count for each system, and each bar is further delineated into the type of protein expressed. Download FIG S4, TIF file, 0.6 MB. Copyright © 2018 Nakajima et al. 2018 Nakajima et al. This content is distributed under the terms of the Creative Commons Attribution 4.0 International license.

10.1128/mSphere.00592-18.5 FIG S5 Signal intensity ratios of proteins expressed in human cell and baculovirus systems. Means of log_2_ signal intensity ratios (+SD) were calculated for 27 HA molecules expressed in both human cell (HC) and baculovirus (BV) expression systems. Briefly, individual log_2_ HC/BV ratios from three groups (natural infection, GCRC, and high-dose H5 boost) were calculated and then averaged for each antigen. Red symbols represent HA1 (head-domain-only) molecules, while the rest are whole HA. Vertical dotted line is at log_2_ = 0, which indicates no change between the two systems. Coefficient of determination (*R*^2^) values of mean signal intensities for human cell-expressed proteins and their corresponding baculovirus-expressed versions are summarized in the table, with *R*^2^ < 0.25 highlighted in beige. Download FIG S5, TIF file, 0.5 MB. Copyright © 2018 Nakajima et al. 2018 Nakajima et al. This content is distributed under the terms of the Creative Commons Attribution 4.0 International license.

10.1128/mSphere.00592-18.6 TABLE S1 List of influenza virus proteins printed on the array. Download Table S1, PDF file, 0.5 MB. Copyright © 2018 Nakajima et al. 2018 Nakajima et al. This content is distributed under the terms of the Creative Commons Attribution 4.0 International license.

**(ii) Microarray probing and development.** Serum samples were diluted 1:100 in protein array blocking buffer (GVS, Sanford, ME, USA) supplemented with E. coli lysate (GenScript, Piscataway, NJ, USA) to a final concentration of 10 mg/ml and preincubated at room temperature (RT) for 30 min. These conditions were established previously to give robust antigen signals while also giving low (<5,000) background signals to E. coli for the majority of seropositive samples. Concurrently, arrays were rehydrated in blocking buffer (without lysate) for 30 min. Blocking buffer was removed, and arrays were probed with preincubated serum samples using sealed chambers to ascertain that there was no cross-contamination of samples between the pads. Arrays were incubated overnight at 4°C with gentle agitation. They were then washed at RT three times with Tris-buffered saline (TBS) containing 0.05% Tween 20 (T-TBS), followed by incubation for 2 h at RT with a mixture of Qdot800-conjugated goat anti-human IgG (Grace Bio-Labs, Inc.) and biotin-conjugated goat anti-human IgA (Jackson ImmunoResearch Laboratories, Inc., West Grove, PA, USA), diluted 1:250 and 1:200, respectively, in blocking buffer. Arrays were washed three times with T-TBS, followed by incubation with streptavidin-conjugated Qdot655 (Thermo Fisher Scientific, Waltham, MA, USA) diluted 1:250 in blocking buffer for 1 h at RT (62). Arrays were washed three times with T-TBS and once with water. Arrays were air dried by centrifugation at 500 × *g* for 10 min. Images were acquired using the ArrayCAM imaging system from Grace Bio-Labs (Bend, OR). Spot and background intensities were measured using an annotated grid (.gal) file. The imager settings were set at gain 50 and 500-ms exposure time for both 655- and 800-nm channels.

### Data analysis.

Purified protein signal intensities used for calculations were first background corrected by subtracting sample-specific T-PBS buffer signals from purified protein spot signals. When the H5 vaccine study is discussed, only data collected from the high-dosage group were used for analysis of antibody responses, unless otherwise indicated. The two-tailed Mann-Whitney test for unpaired data and the two-tailed Kruskal-Wallis test with Dunn’s multiple-comparison test were performed in GraphPad Prism 6 (GraphPad, La Jolla, CA, USA). A *P* value of <0.05 was considered statistically significant. The antigen identifiers and raw data are provided in Table S2 in the supplemental material.

10.1128/mSphere.00592-18.7 TABLE S2 Antigen identifiers and raw protein microarray data probed with naturally exposed and vaccinated serum samples, detected for IgA and IgG antibody responses. Download Table S2, XLSX file, 0.3 MB. Copyright © 2018 Nakajima et al. 2018 Nakajima et al. This content is distributed under the terms of the Creative Commons Attribution 4.0 International license.

For comparisons between low- and high-dosage vaccines, mean fluorescence intensities (MFIs) for all antigens per Ag group (HA1 and HA1+HA2) at t1 and t2 were added per individual sample to obtain an individual’s aggregate reactivity for that time point (for that group of Ags). These were then plotted to compare the dosage groups (high and low). Differences between high- and low-dosage groups were analyzed using the Wilcoxon rank sum test in R, and those between time points were analyzed using the Wilcoxon signed-rank test, where significance was set at *P* < 0.05. Delta increases were calculated by subtracting the MFIs for each antigen t2 − t1 for each volunteer. Delta MFIs for all antigens per antigen group (HA1 and HA1+HA2) were then summed for each individual’s aggregate reactivity for that group of antigens. These were then plotted to compare high- and low-dosage recipients. Differences between high- and low-dosage groups were analyzed using the Wilcoxon rank sum test in R, where significance was set at *P* < 0.05.

## References

[1] Iuliano AD, Roguski KM, Chang HH, Muscatello DJ, Palekar R, Tempia S, Cohen C, Gran JM, Schanzer D, Cowling BJ, Wu P, Kyncl J, Ang LW, Park M, Redlberger-Fritz M, Yu H, Espenhain L, Krishnan A, Emukule G, van Asten L, Pereira da Silva S, Aungkulanon S, Buchholz U, Widdowson M-A, Bresee JS, Azziz-Baumgartner E, Cheng P-Y, Dawood F, Foppa I, Olsen S, Haber M, Jeffers C, MacIntyre CR, for the Global Seasonal Influenza-Associated Mortality Collaborator Network. 2018 Estimates of global seasonal influenza-associated respiratory mortality: a modelling study. Lancet 391:1285–1300. doi:10.1016/S0140-6736(17)33293-2. 29248255 PMC5935243

[2] Smith DJ, Lapedes AS, de Jong JC, Bestebroer TM, Rimmelzwaan GF, Osterhaus AD, Fouchier RA 2004 Mapping the antigenic and genetic evolution of influenza virus. Science 305:371–376. doi:10.1126/science.1097211. 15218094

[3] Koel BF, Burke DF, Bestebroer TM, van der Vliet S, Zondag GC, Vervaet G, Skepner E, Lewis NS, Spronken MI, Russell CA, Eropkin MY, Hurt AC, Barr IG, de Jong JC, Rimmelzwaan GF, Osterhaus AD, Fouchier RA, Smith DJ 2013 Substitutions near the receptor binding site determine major antigenic change during influenza virus evolution. Science 342:976–979. doi:10.1126/science.1244730. 24264991

[4] Fantoni A, Arena C, Corrias L, Salez N, de Lamballerie XN, Amoros JP, Blanchon T, Varesi L, Falchi A 2014 Genetic drift of influenza A(H3N2) viruses during two consecutive seasons in 2011-2013 in Corsica, France. J Med Virol 86:585–591. doi:10.1002/jmv.23745. 24105757

[5] World Health Organization. 2010 Pandemic (H1N1) 2009—update 112. www.who.int/csr/don/2010_08_06/en/index.html.

[6] Simonsen L, Spreeuwenberg P, Lustig R, Taylor RJ, Fleming DM, Kroneman M, Van Kerkhove MD, Mounts AW, Paget WJ, Teams GLC 2013 Global mortality estimates for the 2009 influenza pandemic from the GLaMOR project: a modeling study. PLoS Med 10:e1001558. doi:10.1371/journal.pmed.1001558. 24302890 PMC3841239

[7] Dawood FS, Iuliano AD, Reed C, Meltzer MI, Shay DK, Cheng PY, Bandaranayake D, Breiman RF, Brooks WA, Buchy P, Feikin DR, Fowler KB, Gordon A, Hien NT, Horby P, Huang QS, Katz MA, Krishnan A, Lal R, Montgomery JM, Molbak K, Pebody R, Presanis AM, Razuri H, Steens A, Tinoco YO, Wallinga J, Yu H, Vong S, Bresee J, Widdowson MA 2012 Estimated global mortality associated with the first 12 months of 2009 pandemic influenza A H1N1 virus circulation: a modelling study. Lancet Infect Dis 12:687–695. doi:10.1016/S1473-3099(12)70121-4. 22738893

[8] Potter CW 2001 A history of influenza. J Appl Microbiol 91:572–579. doi:10.1046/j.1365-2672.2001.01492.x. 11576290

[9] Parrish CR, Kawaoka Y 2005 The origins of new pandemic viruses: the acquisition of new host ranges by canine parvovirus and influenza A viruses. Annu Rev Microbiol 59:553–586. doi:10.1146/annurev.micro.59.030804.121059. 16153179

[10] Jennings LC, Monto AS, Chan PK, Szucs TD, Nicholson KG 2008 Stockpiling prepandemic influenza vaccines: a new cornerstone of pandemic preparedness plans. Lancet Infect Dis 8:650–658. doi:10.1016/S1473-3099(08)70232-9. 18922487

[11] Ekiert DC, Bhabha G, Elsliger MA, Friesen RH, Jongeneelen M, Throsby M, Goudsmit J, Wilson IA 2009 Antibody recognition of a highly conserved influenza virus epitope. Science 324:246–251. doi:10.1126/science.1171491. 19251591 PMC2758658

[12] Wiley DC, Wilson IA, Skehel JJ 1981 Structural identification of the antibody-binding sites of Hong Kong influenza haemagglutinin and their involvement in antigenic variation. Nature 289:373–378. doi:10.1038/289373a0. 6162101

[13] Knossow M, Daniels RS, Douglas AR, Skehel JJ, Wiley DC 1984 Three-dimensional structure of an antigenic mutant of the influenza virus haemagglutinin. Nature 311:678–680. doi:10.1038/311678a0. 6207440

[14] Luoh SM, McGregor MW, Hinshaw VS 1992 Hemagglutinin mutations related to antigenic variation in H1 swine influenza viruses. J Virol 66:1066–1073. 1731091 10.1128/jvi.66.2.1066-1073.1992 PMC240810

[15] Krammer F, Palese P 2015 Advances in the development of influenza virus vaccines. Nat Rev Drug Discov 14:167–182. doi:10.1038/nrd4529. 25722244

[16] Pica N, Palese P 2013 Toward a universal influenza virus vaccine: prospects and challenges. Annu Rev Med 64:189–202. doi:10.1146/annurev-med-120611-145115. 23327522

[17] Cho A, Wrammert J 2016 Implications of broadly neutralizing antibodies in the development of a universal influenza vaccine. Curr Opin Virol 17:110–115. doi:10.1016/j.coviro.2016.03.002. 27031684 PMC4940123

[18] Corti D, Lanzavecchia A 2013 Broadly neutralizing antiviral antibodies. Annu Rev Immunol 31:705–742. doi:10.1146/annurev-immunol-032712-095916. 23330954

[19] Sui J, Hwang WC, Perez S, Wei G, Aird D, Chen LM, Santelli E, Stec B, Cadwell G, Ali M, Wan H, Murakami A, Yammanuru A, Han T, Cox NJ, Bankston LA, Donis RO, Liddington RC, Marasco WA 2009 Structural and functional bases for broad-spectrum neutralization of avian and human influenza A viruses. Nat Struct Mol Biol 16:265–273. doi:10.1038/nsmb.1566. 19234466 PMC2692245

[20] Joyce MG, Wheatley AK, Thomas PV, Chuang GY, Soto C, Bailer RT, Druz A, Georgiev IS, Gillespie RA, Kanekiyo M, Kong WP, Leung K, Narpala SN, Prabhakaran MS, Yang ES, Zhang B, Zhang Y, Asokan M, Boyington JC, Bylund T, Darko S, Lees CR, Ransier A, Shen CH, Wang L, Whittle JR, Wu X, Yassine HM, Santos C, Matsuoka Y, Tsybovsky Y, Baxa U, Program NCS, Mullikin JC, Subbarao K, Douek DC, Graham BS, Koup RA, Ledgerwood JE, Roederer M, Shapiro L, Kwong PD, Mascola JR, McDermott AB 2016 Vaccine-induced antibodies that neutralize group 1 and group 2 influenza A viruses. Cell 166:609–623. doi:10.1016/j.cell.2016.06.043. 27453470 PMC4978566

[21] Kallewaard NL, Corti D, Collins PJ, Neu U, McAuliffe JM, Benjamin E, Wachter-Rosati L, Palmer-Hill FJ, Yuan AQ, Walker PA, Vorlaender MK, Bianchi S, Guarino B, De Marco A, Vanzetta F, Agatic G, Foglierini M, Pinna D, Fernandez-Rodriguez B, Fruehwirth A, Silacci C, Ogrodowicz RW, Martin SR, Sallusto F, Suzich JA, Lanzavecchia A, Zhu Q, Gamblin SJ, Skehel JJ 2016 Structure and function analysis of an antibody recognizing all influenza A subtypes. Cell 166:596–608. doi:10.1016/j.cell.2016.05.073. 27453466 PMC4967455

[22] Krammer F, Palese P, Steel J 2015 Advances in universal influenza virus vaccine design and antibody mediated therapies based on conserved regions of the hemagglutinin. Curr Top Microbiol Immunol 386:301–321. doi:10.1007/82_2014_408. 25007847

[23] Doyle TM, Hashem AM, Li C, Van Domselaar G, Larocque L, Wang J, Smith D, Cyr T, Farnsworth A, He R, Hurt AC, Brown EG, Li X 2013 Universal anti-neuraminidase antibody inhibiting all influenza A subtypes. Antiviral Res 100:567–574. doi:10.1016/j.antiviral.2013.09.018. 24091204

[24] Wohlbold TJ, Nachbagauer R, Xu H, Tan GS, Hirsh A, Brokstad KA, Cox RJ, Palese P, Krammer F 2015 Vaccination with adjuvanted recombinant neuraminidase induces broad heterologous, but not heterosubtypic, cross-protection against influenza virus infection in mice. mBio 6:e02556. doi:10.1128/mBio.02556-14. 25759506 PMC4453582

[25] Wrammert J, Koutsonanos D, Li GM, Edupuganti S, Sui J, Morrissey M, McCausland M, Skountzou I, Hornig M, Lipkin WI, Mehta A, Razavi B, Del Rio C, Zheng NY, Lee JH, Huang M, Ali Z, Kaur K, Andrews S, Amara RR, Wang Y, Das SR, O’Donnell CD, Yewdell JW, Subbarao K, Marasco WA, Mulligan MJ, Compans R, Ahmed R, Wilson PC 2011 Broadly cross-reactive antibodies dominate the human B cell response against 2009 pandemic H1N1 influenza virus infection. J Exp Med 208:181–193. doi:10.1084/jem.20101352. 21220454 PMC3023136

[26] Zhao H, Fernandez E, Dowd KA, Speer SD, Platt DJ, Gorman MJ, Govero J, Nelson CA, Pierson TC, Diamond MS, Fremont DH 2016 Structural basis of Zika virus-specific antibody protection. Cell 166:1016–1027. doi:10.1016/j.cell.2016.07.020. 27475895 PMC4983199

[27] Sapparapu G, Fernandez E, Kose N, Bin C, Fox JM, Bombardi RG, Zhao H, Nelson CA, Bryan AL, Barnes T, Davidson E, Mysorekar IU, Fremont DH, Doranz BJ, Diamond MS, Crowe JE 2016 Neutralizing human antibodies prevent Zika virus replication and fetal disease in mice. Nature 540:443–447. doi:10.1038/nature20564. 27819683 PMC5583716

[28] Scheid JF, Mouquet H, Feldhahn N, Seaman MS, Velinzon K, Pietzsch J, Ott RG, Anthony RM, Zebroski H, Hurley A, Phogat A, Chakrabarti B, Li Y, Connors M, Pereyra F, Walker BD, Wardemann H, Ho D, Wyatt RT, Mascola JR, Ravetch JV, Nussenzweig MC 2009 Broad diversity of neutralizing antibodies isolated from memory B cells in HIV-infected individuals. Nature 458:636–640. doi:10.1038/nature07930. 19287373

[29] Desbien AL, Van Hoeven N, Reed SJ, Casey AC, Laurance JD, Baldwin SL, Duthie MS, Reed SG, Carter D 2013 Development of a high density hemagglutinin protein microarray to determine the breadth of influenza antibody responses. Biotechniques 54:345–348. doi:10.2144/000114041. 23750544

[30] Koopmans M, de Bruin E, Godeke GJ, Friesema I, van Gageldonk R, Schipper M, Meijer A, van Binnendijk R, Rimmelzwaan GF, de Jong MD, Buisman A, van Beek J, van de Vijver D, Reimerink J 2012 Profiling of humoral immune responses to influenza viruses by using protein microarray. Clin Microbiol Infect 18:797–807. doi:10.1111/j.1469-0691.2011.03701.x. 22212116

[31] Te Beest DE, de Bruin E, Imholz S, Koopmans M, van Boven M 2017 Heterosubtypic cross-reactivity of HA1 antibodies to influenza A, with emphasis on nonhuman subtypes (H5N1, H7N7, H9N2). PLoS One 12:e0181093. doi:10.1371/journal.pone.0181093. 28715468 PMC5513445

[32] Freidl GS, de Bruin E, van Beek J, Reimerink J, de Wit S, Koch G, Vervelde L, van den Ham HJ, Koopmans MP 2014 Getting more out of less—a quantitative serological screening tool for simultaneous detection of multiple influenza A hemagglutinin-types in chickens. PLoS One 9:e108043. doi:10.1371/journal.pone.0108043. 25248105 PMC4172590

[33] Price JV, Jarrell JA, Furman D, Kattah NH, Newell E, Dekker CL, Davis MM, Utz PJ 2013 Characterization of influenza vaccine immunogenicity using influenza antigen microarrays. PLoS One 8:e64555. doi:10.1371/journal.pone.0064555. 23734205 PMC3667171

[34] Winokur PL, Patel SM, Brady R, Chen WH, El-Kamary SS, Edwards K, Creech CB, Frey S, Keitel WA, Belshe R, Walter E, Bellamy A, Hill H 2015 Safety and immunogenicity of a single low dose or high dose of clade 2 influenza A(H5N1) inactivated vaccine in adults previously primed with clade 1 influenza A(H5N1) vaccine. J Infect Dis 212:525–530. doi:10.1093/infdis/jiv087. 25712967 PMC4598805

[35] Khurana S, Coyle EM, Dimitrova M, Castellino F, Nicholson K, Del Giudice G, Golding H 2014 Heterologous prime-boost vaccination with MF59-adjuvanted H5 vaccines promotes antibody affinity maturation towards the hemagglutinin HA1 domain and broad H5N1 cross-clade neutralization. PLoS One 9:e95496. doi:10.1371/journal.pone.0095496. 24755693 PMC3995799

[36] Gillard P, Chu DW, Hwang SJ, Yang PC, Thongcharoen P, Lim FS, Drame M, Walravens K, Roman F 2014 Long-term booster schedules with AS03A-adjuvanted heterologous H5N1 vaccines induces rapid and broad immune responses in Asian adults. BMC Infect Dis 14:142. doi:10.1186/1471-2334-14-142. 24628789 PMC4008266

[37] Centers for Disease Control and Prevention. 2011 2010–2011 influenza (flu) season. www.cdc.gov/flu/pastseasons/1011season.htm.

[38] Robertson JS, Bootman JS, Newman R, Oxford JS, Daniels RS, Webster RG, Schild GC 1987 Structural changes in the haemagglutinin which accompany egg adaptation of an influenza A(H1N1) virus. Virology 160:31–37. doi:10.1016/0042-6822(87)90040-7. 3629978

[39] Gambaryan AS, Marinina VP, Tuzikov AB, Bovin NV, Rudneva IA, Sinitsyn BV, Shilov AA, Matrosovich MN 1998 Effects of host-dependent glycosylation of hemagglutinin on receptor-binding properties on H1N1 human influenza A virus grown in MDCK cells and in embryonated eggs. Virology 247:170–177. doi:10.1006/viro.1998.9224. 9705910

[40] Katz JM, Wang M, Webster RG 1990 Direct sequencing of the HA gene of influenza (H3N2) virus in original clinical samples reveals sequence identity with mammalian cell-grown virus. J Virol 64:1808–1811. 2319652 10.1128/jvi.64.4.1808-1811.1990 PMC249319

[41] Meade P, Latorre-Margalef N, Stallknecht DE, Krammer F 2017 Development of an influenza virus protein microarray to measure the humoral response to influenza virus infection in mallards. Emerg Microbes Infect 6:e110. doi:10.1038/emi.2017.98. 29209053 PMC5750464

[42] de Vries RD, Altenburg AF, Nieuwkoop NJ, de Bruin E, van Trierum SE, Pronk MR, Lamers MM, Richard M, Nieuwenhuijse DF, Koopmans MPG, Kreijtz J, Fouchier RAM, Osterhaus A, Sutter G, Rimmelzwaan GF 2018 Induction of cross-clade antibody and T-cell responses by a modified vaccinia virus Ankara-based influenza A(H5N1) vaccine in a randomized phase 1/2a clinical trial. J Infect Dis 218:614–623. doi:10.1093/infdis/jiy214. 29912453 PMC6047453

[43] Hoft DF, Lottenbach K, Goll JB, Hill H, Winokur PL, Patel SM, Brady RC, Chen WH, Edwards K, Creech CB, Frey SE, Blevins TP, Salomon R, Belshe RB 2016 Priming vaccination with influenza virus H5 hemagglutinin antigen significantly increases the duration of T cell responses induced by a heterologous H5 booster vaccination. J Infect Dis 214:1020–1029. doi:10.1093/infdis/jiw310. 27443611 PMC5021235

[44] Ellebedy AH, Krammer F, Li GM, Miller MS, Chiu C, Wrammert J, Chang CY, Davis CW, McCausland M, Elbein R, Edupuganti S, Spearman P, Andrews SF, Wilson PC, García-Sastre A, Mulligan MJ, Mehta AK, Palese P, Ahmed R 2014 Induction of broadly cross-reactive antibody responses to the influenza HA stem region following H5N1 vaccination in humans. Proc Natl Acad Sci U S A 111:13133–13138. doi:10.1073/pnas.1414070111. 25157133 PMC4246941

[45] Nachbagauer R, Wohlbold TJ, Hirsh A, Hai R, Sjursen H, Palese P, Cox RJ, Krammer F 2014 Induction of broadly reactive anti-hemagglutinin stalk antibodies by an H5N1 vaccine in humans. J Virol 88:13260–13268. doi:10.1128/JVI.02133-14. 25210189 PMC4249097

[46] Nachbagauer R, Choi A, Hirsh A, Margine I, Iida S, Barrera A, Ferres M, Albrecht RA, Garcia-Sastre A, Bouvier NM, Ito K, Medina RA, Palese P, Krammer F 2017 Defining the antibody cross-reactome directed against the influenza virus surface glycoproteins. Nat Immunol 18:464–473. doi:10.1038/ni.3684. 28192418 PMC5360498

[47] Mbawuike IN, Pacheco S, Acuna CL, Switzer KC, Zhang Y, Harriman GR 1999 Mucosal immunity to influenza without IgA: an IgA knockout mouse model. J Immunol 162:2530–2537. 10072492

[48] Benton KA, Misplon JA, Lo CY, Brutkiewicz RR, Prasad SA, Epstein SL 2001 Heterosubtypic immunity to influenza A virus in mice lacking IgA, all Ig, NKT cells, or gamma delta T cells. J Immunol 166:7437–7445. doi:10.4049/jimmunol.166.12.7437. 11390496

[49] Renegar KB, Johnson CD, Dewitt RC, King BK, Li J, Fukatsu K, Kudsk KA 2001 Impairment of mucosal immunity by total parenteral nutrition: requirement for IgA in murine nasotracheal anti-influenza immunity. J Immunol 166:819–825. doi:10.4049/jimmunol.166.2.819. 11145655

[50] Arulanandam BP, Raeder RH, Nedrud JG, Bucher DJ, Le J, Metzger DW 2001 IgA immunodeficiency leads to inadequate Th cell priming and increased susceptibility to influenza virus infection. J Immunol 166:226–231. doi:10.4049/jimmunol.166.1.226. 11123296

[51] Brandtzaeg P 2003 Role of mucosal immunity in influenza. Dev Biol (Basel) 115:39–48. 15088774

[52] Belshe R, Lee MS, Walker RE, Stoddard J, Mendelman PM 2004 Safety, immunogenicity and efficacy of intranasal, live attenuated influenza vaccine. Expert Rev Vaccines 3:643–654. doi:10.1586/14760584.3.6.643. 15606348

[53] Tamura S, Ainai A, Suzuki T, Kurata T, Hasegawa H 2016 Intranasal inactivated influenza vaccines: a reasonable approach to improve the efficacy of influenza vaccine? Jpn J Infect Dis 69:165–179. doi:10.7883/yoken.JJID.2015.560. 27212584

[54] van Riet E, Ainai A, Suzuki T, Hasegawa H 2012 Mucosal IgA responses in influenza virus infections; thoughts for vaccine design. Vaccine 30:5893–5900. doi:10.1016/j.vaccine.2012.04.109. 22835738

[55] Margine I, Hai R, Albrecht RA, Obermoser G, Harrod AC, Banchereau J, Palucka K, Garcia-Sastre A, Palese P, Treanor JJ, Krammer F 2013 H3N2 influenza virus infection induces broadly reactive hemagglutinin stalk antibodies in humans and mice. J Virol 87:4728–4737. doi:10.1128/JVI.03509-12. 23408625 PMC3624338

[56] Henry Dunand CJ, Leon PE, Huang M, Choi A, Chromikova V, Ho IY, Tan GS, Cruz J, Hirsh A, Zheng NY, Mullarkey CE, Ennis FA, Terajima M, Treanor JJ, Topham DJ, Subbarao K, Palese P, Krammer F, Wilson PC 2016 Both neutralizing and non-neutralizing human H7N9 influenza vaccine-induced monoclonal antibodies confer protection. Cell Host Microbe 19:800–813. doi:10.1016/j.chom.2016.05.014. 27281570 PMC4901526

[57] Tan GS, Leon PE, Albrecht RA, Margine I, Hirsh A, Bahl J, Krammer F 2016 Broadly-reactive neutralizing and non-neutralizing antibodies directed against the H7 influenza virus hemagglutinin reveal divergent mechanisms of protection. PLoS Pathog 12:e1005578. doi:10.1371/journal.ppat.1005578. 27081859 PMC4833315

[58] Lee J, Boutz DR, Chromikova V, Joyce MG, Vollmers C, Leung K, Horton AP, DeKosky BJ, Lee CH, Lavinder JJ, Murrin EM, Chrysostomou C, Hoi KH, Tsybovsky Y, Thomas PV, Druz A, Zhang B, Zhang Y, Wang L, Kong WP, Park D, Popova LI, Dekker CL, Davis MM, Carter CE, Ross TM, Ellington AD, Wilson PC, Marcotte EM, Mascola JR, Ippolito GC, Krammer F, Quake SR, Kwong PD, Georgiou G 2016 Molecular-level analysis of the serum antibody repertoire in young adults before and after seasonal influenza vaccination. Nat Med 22:1456–1464. doi:10.1038/nm.4224. 27820605 PMC5301914

[59] Oshansky CM, Gartland AJ, Wong SS, Jeevan T, Wang D, Roddam PL, Caniza MA, Hertz T, Devincenzo JP, Webby RJ, Thomas PG 2014 Mucosal immune responses predict clinical outcomes during influenza infection independently of age and viral load. Am J Respir Crit Care Med 189:449–462. doi:10.1164/rccm.201309-1616OC. 24308446 PMC3977720

[60] Centers for Disease Control and Prevention. 2008 Flu season summary (September 30, 2007 - May 17, 2008). www.cdc.gov/flu/pastseasons/0708season.htm.

[61] Van Hoeven N, Fox CB, Granger B, Evers T, Joshi SW, Nana GI, Evans SC, Lin S, Liang H, Liang L, Nakajima R, Felgner PL, Bowen RA, Marlenee N, Hartwig A, Baldwin SL, Coler RN, Tomai M, Elvecrog J, Reed SG, Carter D 2017 A formulated TLR7/8 agonist is a flexible, highly potent and effective adjuvant for pandemic influenza vaccines. Sci Rep 7:46426. doi:10.1038/srep46426. 28429728 PMC5399443

[62] Jain A, Taghavian O, Vallejo D, Dotsey E, Schwartz D, Bell FG, Greef C, Davies DH, Grudzien J, Lee AP, Felgner PL, Liang L 2016 Evaluation of quantum dot immunofluorescence and a digital CMOS imaging system as an alternative to conventional organic fluorescence dyes and laser scanning for quantifying protein microarrays. Proteomics 16:1271–1279. doi:10.1002/pmic.201500375. 26842269 PMC4973892

