# Supplementary figures and images for: Protein Microarray Analysis of the Specificity and Cross-Reactivity of Influenza Virus Hemagglutinin-Specific Antibodies
Source: mSphere. 2018 Dec 12;3(6):e00592-18. doi: 10.1128/mSphere.00592-18 (PMC6291623; doi:10.1128/mSphere.00592-18)

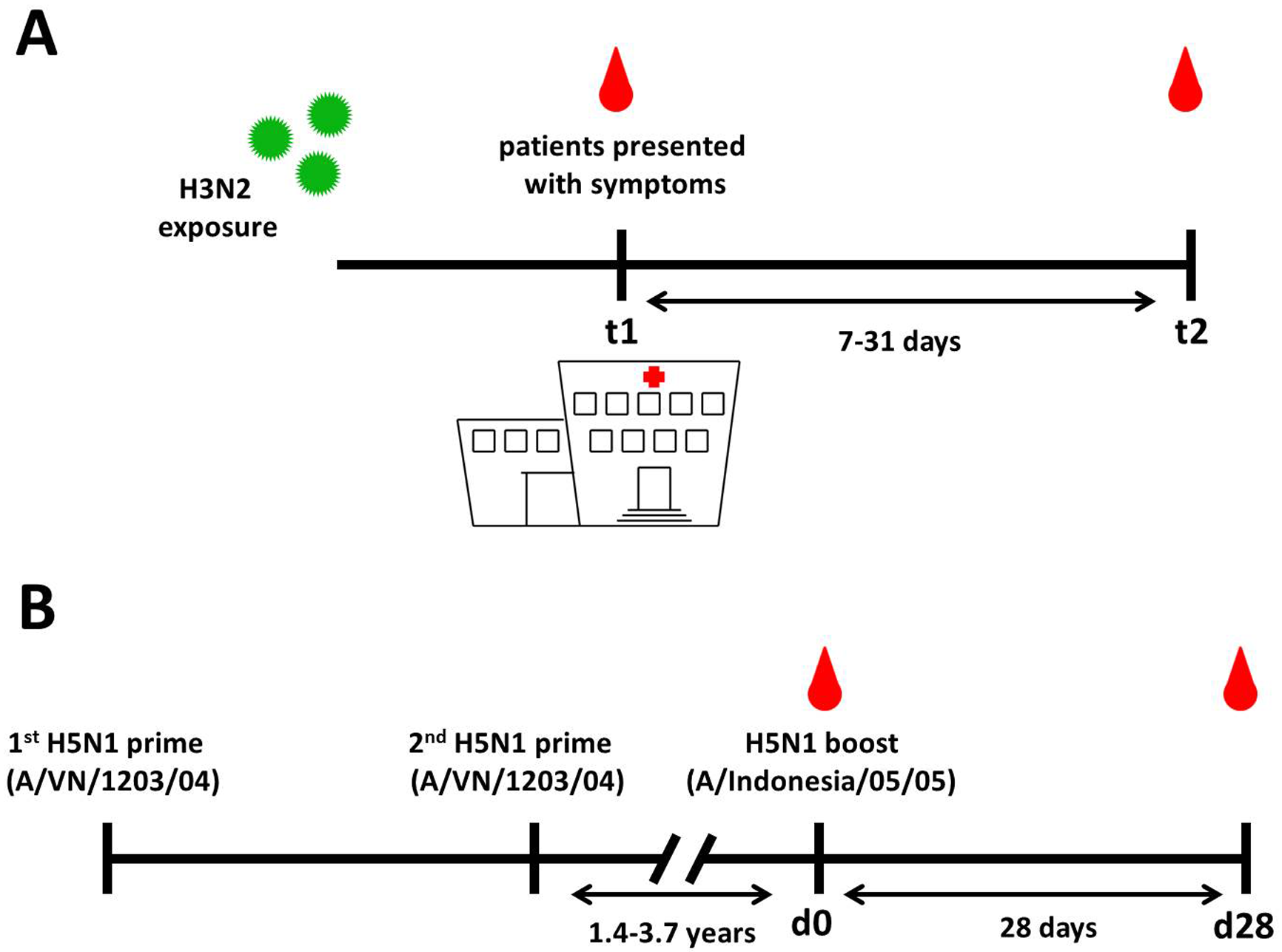

Supplement: FIG S1 [file sph006182722sf1.tif]

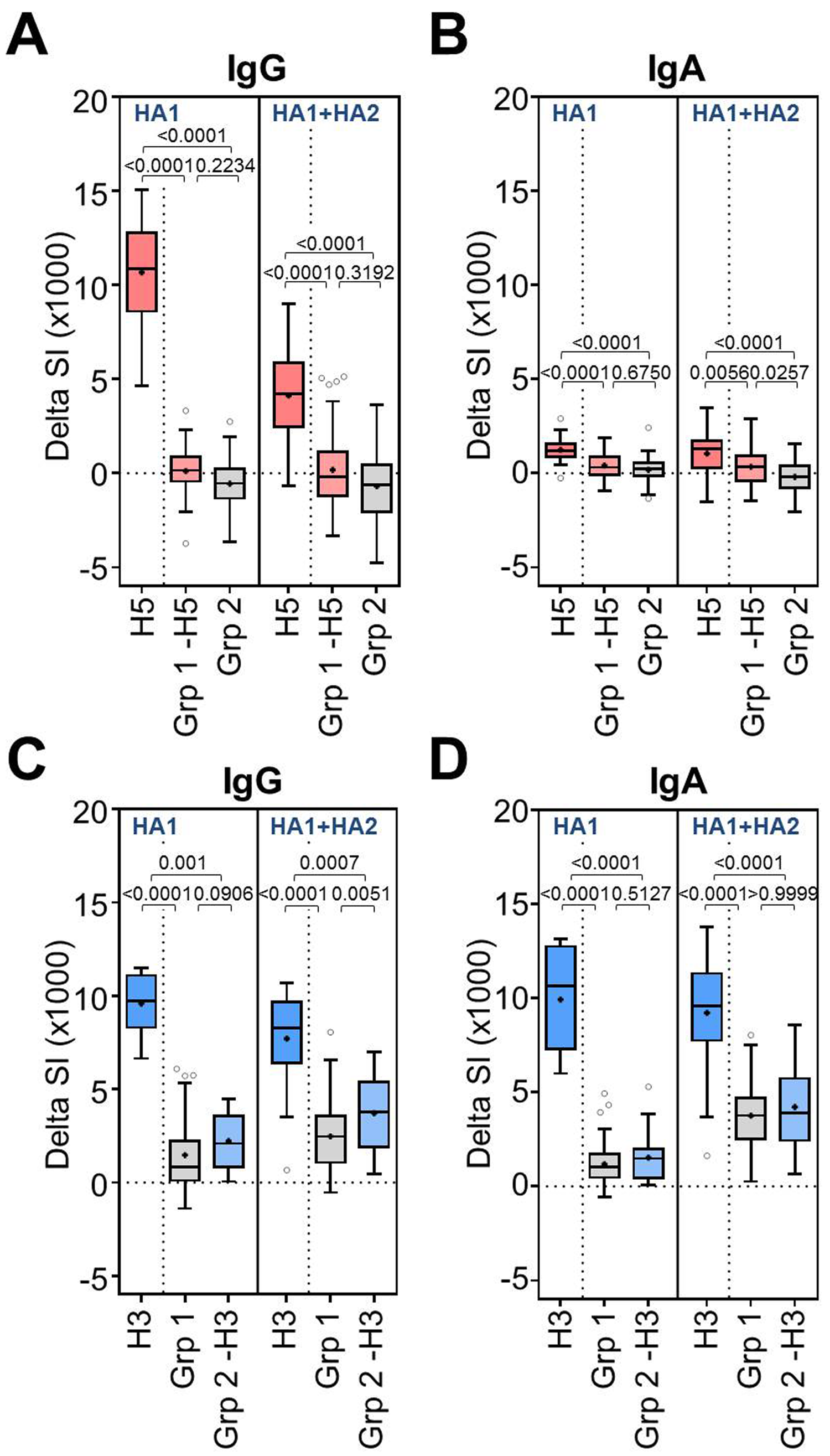

Supplement: FIG S2 [file sph006182722sf2.tif]

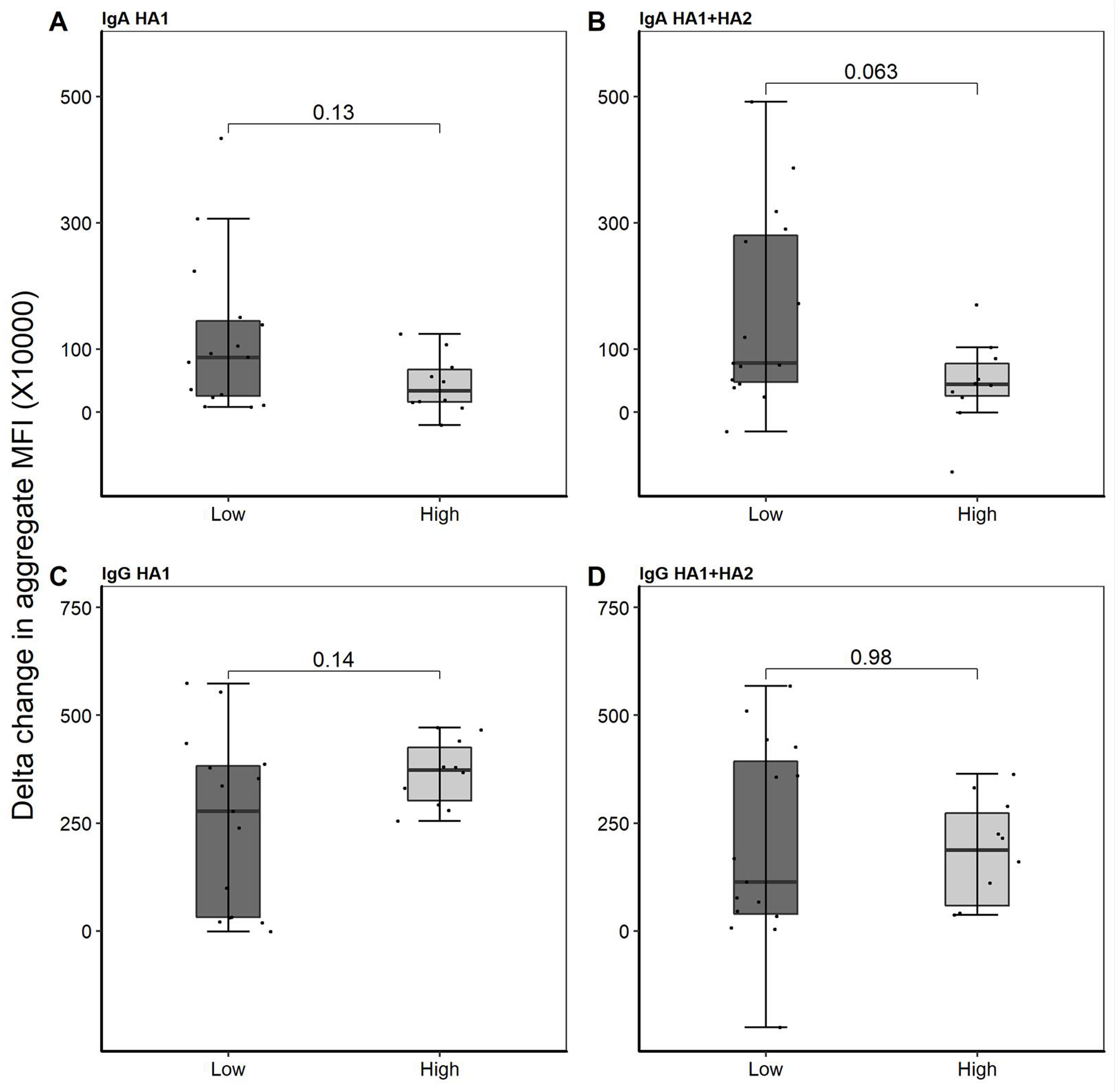

Supplement: FIG S3 [file sph006182722sf3.tif]

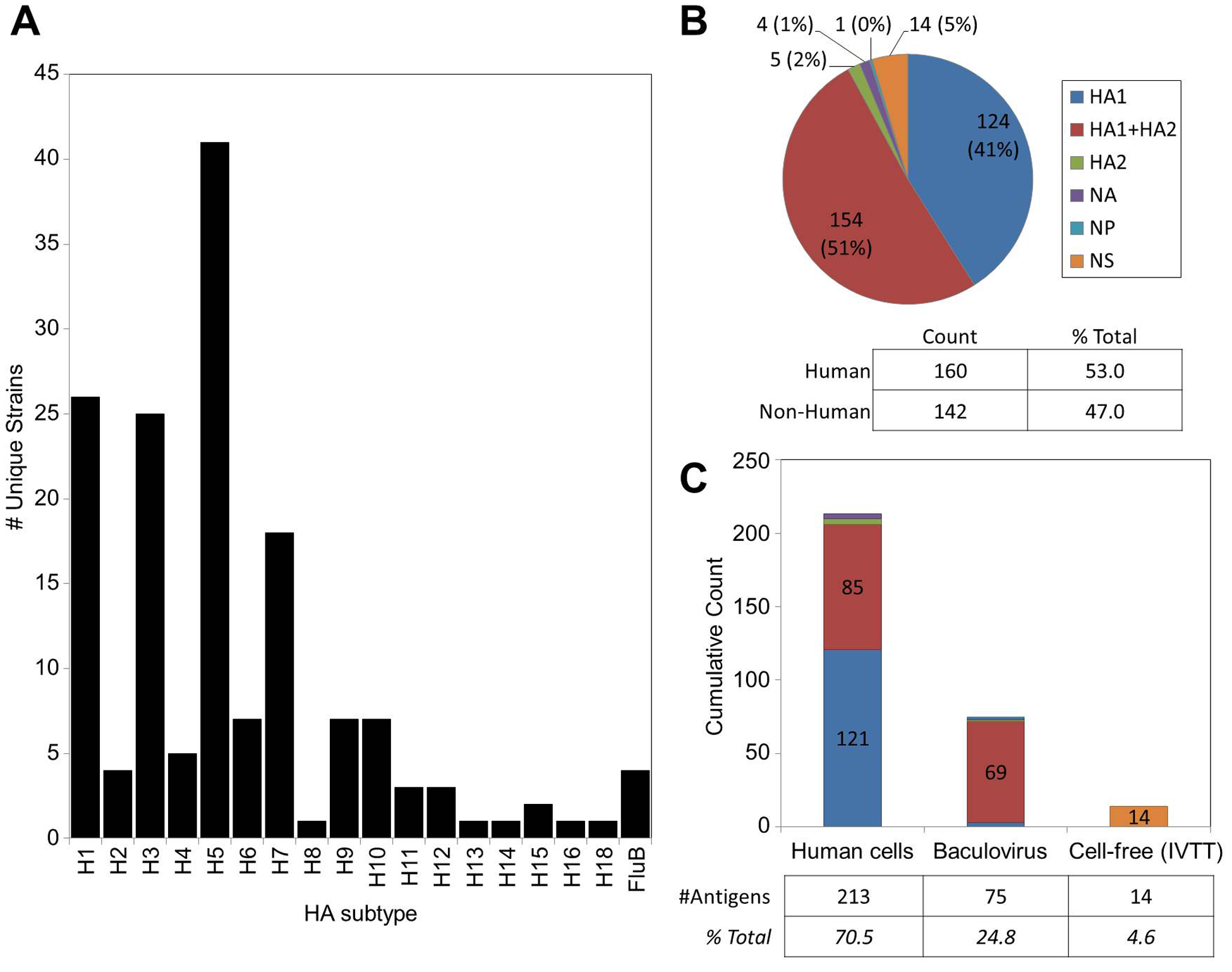

Supplement: FIG S4 [file sph006182722sf4.tif]

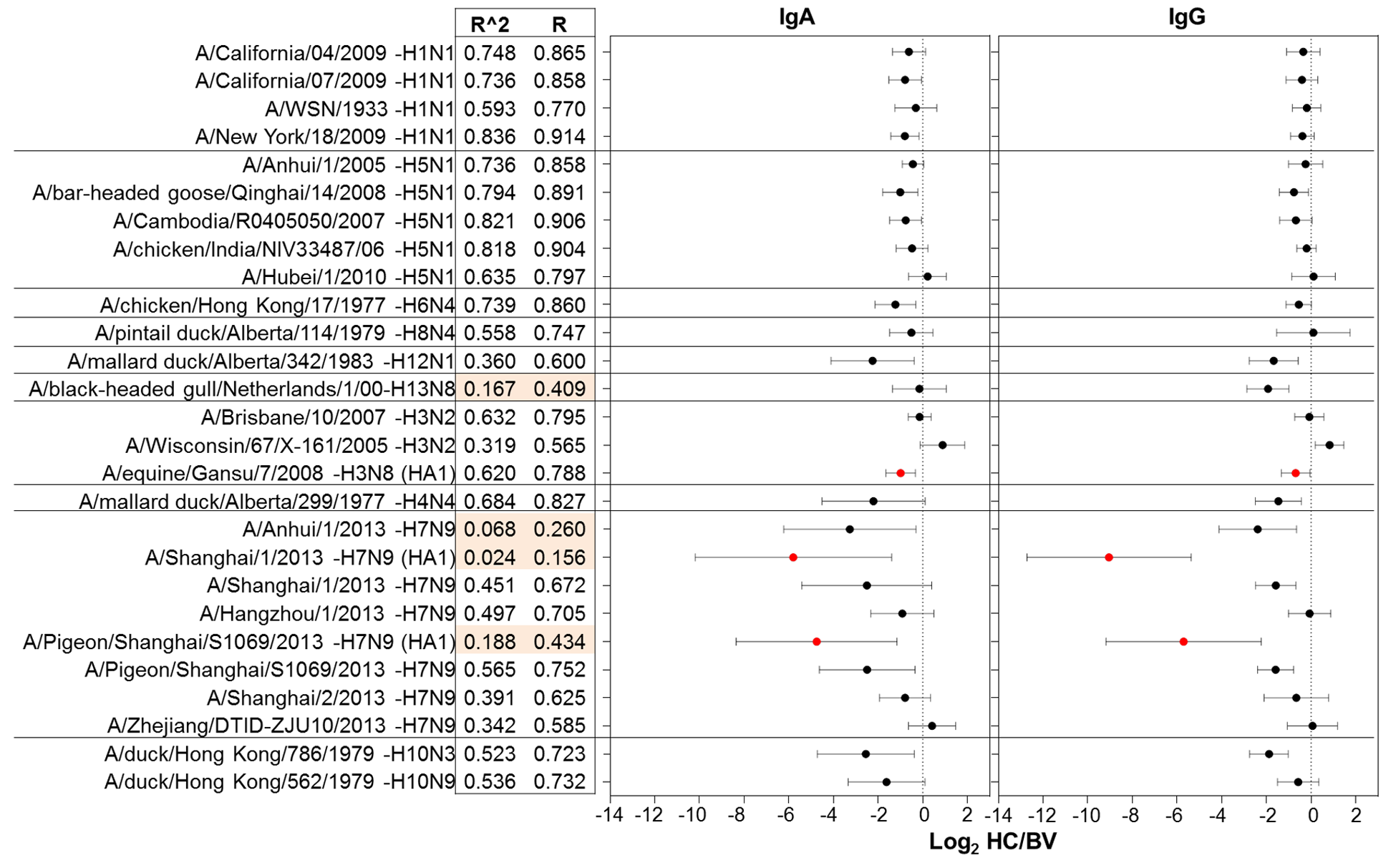

Supplement: FIG S5 [file sph006182722sf5.tif]
