# Supplementary material for: Protein Microarray Analysis of the Specificity and Cross-Reactivity of Influenza Virus Hemagglutinin-Specific Antibodies
Source: mSphere. 2018 Dec 12;3(6):e00592-18. doi: 10.1128/mSphere.00592-18 (PMC6291623; doi:10.1128/mSphere.00592-18)
Supplement: TABLE S1 [file sph006182722st1.pdf]

Cat no.	Strain	Type	Subtype	Clade	Group	Molecule[a]	Cell Express. System	Tag[b]	GenBank[c]
40035-V08H1	A/Beijing/22808/2009	A	H1N1		1	HA1	Human	His	ABI84516.1
40035-V08H	A/Beijing/22808/2009	A	H1N1		1	HA1+ HA2	Human	His	ADD64203.1
40133-V08B	A/Beijing/262/1995	A	H1N1		1	HA1+ HA2	Baculo-virus	His	ACF41867.1
11068-V08H1	A/Brevig Mission/1/1918	A	H1N1		1	HA1	Human	His	AAD17229.1
11068-V08H	A/Brevig Mission/1/1918	A	H1N1		1	HA1+ HA2	Human	His	AAD17229.1
11052-V08H1	A/Brisbane/59/2007	A	H1N1		1	HA1	Human	His	ACA28844.1
11052-V08H	A/Brisbane/59/2007	A	H1N1		1	HA1+ HA2	Human	His	ACA28844.1
11055-V08H4	A/California/04/2009	A	H1N1		1	HA1	Human	His	ACP41105.1
11055-V08H2	A/California/04/2009	A	H1N1		1	HA1+ HA2	Human	His	ACP41105.1
11055-VNAB	A/California/04/2009	A	H1N1		1	HA1+ HA2 (HA0)	Baculo-virus	n.s.	n.s.
11055-V04H3	A/California/04/2009	A	H1N1		1	HA2	Human	Fc (mo)	ACP41105.1
11055-V08B	A/California/04/2009	A	H1N1		1	HA1+ HA2	Baculo-virus	His	ACP41105.1
11055-V08H	A/California/04/2009	A	H1N1		1	HA1+ HA2	Human	His	ACP41105.1
40350-V08H1	A/California/06/2009	A	H1N1		1	HA1	Human	His	ACP41935.1
11085-V08B	A/California/07/2009	A	H1N1		1	HA1+ HA2	Baculo-virus	His	ACP41953.1
11085-V08H	A/California/07/2009	A	H1N1		1	HA1+ HA2	Human	His	ACP44189.1
40005-V08H1	A/England/195/2009	A	H1N1		1	HA1	Human	His	ACR15621.1
40005-V08H	A/England/195/2009	A	H1N1		1	HA1+ HA2	Human	His	ACR15621.1
11683-V08H1	A/New Caledonia/20/99	A	H1N1		1	HA1	Human	His	AAP34324.1
11683-V08H	A/New Caledonia/20/99	A	H1N1		1	HA1+ HA2	Human	His	AAP34324.1
40090-V08H1	A/New York/1/1918	A	H1N1		1	HA1	Human	His	AAD17219.1
40090-V08B	A/New York/1/1918	A	H1N1		1	HA1+ HA2	Baculo-virus	His	AAD17219.1
40009-V08H1	A/New York/18/2009	A	H1N1		1	HA1	Human	His	ACU13097.1
40009-V08B	A/New York/18/2009	A	H1N1		1	HA1+ HA2	Baculo-virus	His	ACR08536.1
40009-V08H	A/New York/18/2009	A	H1N1		1	HA1+ HA2	Human	His	ACU13097.1
40007-V08H1	A/Ohio/07/2009	A	H1N1		1	HA1	Human	His	ACR38870.1

40007-V08H	A/Ohio/07/2009	A	H1N1		1	HA1+ HA2	Human	His	ACQ63286.1
11687-V08H1	A/Ohio/UR06-0091/2007	A	H1N1		1	HA1	Human	His	ABW40422.1
11687-V08H	A/Ohio/UR06-0091/2007	A	H1N1		1	HA1+ HA2	Human	His	ABW40422.1
11684-V08H1	A/Puerto Rico/8/34	A	H1N1		1	HA1	Human	His	ABD77675.1
11684-V08H	A/Puerto Rico/8/34	A	H1N1		1	HA1+ HA2	Human	His	ABD77675.1
11708-V08H1	A/Solomon Islands/3/2006	A	H1N1		1	HA1	Human	His	ABU99109.1
11708-V08H	A/Solomon Islands/3/2006	A	H1N1		1	HA1+ HA2	Human	His	ABU99109.1
40131-V08B	A/Taiwan/01/1986	A	H1N1		1	HA1+ HA2	Baculo-virus	His	ABF21274.1
40006-V08H1	A/Texas/05/2009	A	H1N1		1	HA1	Human	His	ACP41934.1
40006-V08H	A/Texas/05/2009	A	H1N1		1	HA1+ HA2	Human	His	ACP41934.1
40132-V08H1	A/Texas/36/1991	A	H1N1		1	HA1	Human	His	ACF41933.1
40134-V08H1	A/USSR/90/1977	A	H1N1		1	HA1	Human	His	P03453
40134-V08B	A/USSR/90/1977	A	H1N1		1	HA1+ HA2	Baculo-virus	His	P03453.2
11692-V08H1	A/WSN/1933	A	H1N1		1	HA1	Human	His	ACF54598.1
11692-V08B	A/WSN/1933	A	H1N1		1	HA1+ HA2	Baculo-virus	His	ACF54598.1
11692-V08H	A/WSN/1933	A	H1N1		1	HA1+ HA2	Human	His	ACF54598.1
pMG107	A/Swine/Iowa/15/1930	A	H1N1		1	NS	Cell-free	His	n.s.
pSN113	A/Swine/England/283902/1993	A	H1N1		1	NS	Cell-free	His	n.s.
pSN117	A/California/04/2009	A	H1N1 (pdm)		1	NS	Cell-free	His	n.s.
11703-V08H1	A/swine/Guangxi/13/2006	A	H1N2		1	HA1	Human	His	ABQ42444.1
11703-V08H	A/swine/Guangxi/13/2006	A	H1N2		1	HA1+ HA2	Human	His	ABQ42444.1
11685-V08H1	A/duck/NZL/160/1976	A	H1N3		1	HA1	Human	His	ABB20429.1
11685-V08H	A/duck/NZL/160/1976	A	H1N3		1	HA1+ HA2	Human	His	ABB20429.1
40135-V08H1	A/Egyptian goose/South Africa/AI1448/2007	A	H1N8		1	HA1	Human	His	n.s.
40136-V08H1	A/mallard/Ohio/265/1987	A	H1N9		1	HA1	Human	His	ABK40634.1
40136-V08B	A/mallard/Ohio/265/1987	A	H1N9		1	HA1+ HA2	Baculo-virus	His	ABK40634.1
11688-V08H1	A/Canada/720/2005	A	H2N2		1	HA1	Human	His	AAY28987.1
11688-V08H	A/Canada/720/2005	A	H2N2		1	HA1+ HA2	Human	His	AAY28987.1
40119-V08H1	A/Guiyang/1/1957	A	H2N2		1	HA1	Human	His	ACD85231.1

40119-V08B	A/Guiyang/1/1957	A	H2N2		1	HA1+ HA2	Baculo-virus	His	ACD85231.1
11088-V08H1	A/Japan/305/1957	A	H2N2		1	HA1	Human	His	AAO46269.1
11088-V08H	A/Japan/305/1957	A	H2N2		1	HA1+ HA2	Human	His	AAA43185.1
pSN109	A/Albany/2/1958	A	H2N2		1	NS	Cell-free	His	n.s.
11048-V08H2	A/Anhui/1/2005	A	H5N1	2.3.4	1	HA1	Human	His	ABD28180.1
11048-VNAH2	A/Anhui/1/2005	A	H5N1	2.3.4	1	HA1	Human	n.s.	ABD28180.1
11048-V06H1	A/Anhui/1/2005	A	H5N1	2.3.4	1	HA1+ HA2	Human	His+ Fc (mo)	ABD28180.1
11048-V08H4	A/Anhui/1/2005	A	H5N1	2.3.4	1	HA1+ HA2	Human	His	ABD28180.1
11048-V08B	A/Anhui/1/2005	A	H5N1	2.3.4	1	HA1+ HA2	Baculo-virus	His	ABD28180.1
11048-V08H1	A/Anhui/1/2005	A	H5N1	2.3.4	1	HA1+ HA2	Human	His	ABD28180.1
11059-V08H2	A/bar-headed goose/Qinghai/14/2008	A	H5N1	0	1	HA1+ HA2	Human	His	ACL28277.1
11059-V08B1	A/bar-headed goose/Qinghai/14/2008	A	H5N1	0	1	HA1+ HA2	Baculo-virus	His	ACL28277.1
11059-V08H1	A/bar-headed goose/Qinghai/14/2008	A	H5N1	0	1	HA1+ HA2	Human	His	ACL28277.1
40117-V08H1	A/bar-headed goose/Qinghai/1A/2005	A	H5N1	2.2	1	HA1	Human	His	ABF93441.1
40117-V08B	A/bar-headed goose/Qinghai/1A/2005	A	H5N1	2.2	1	HA1+ HA2	Baculo-virus	His	ABF93441.1
40160-V08H1	A/barn swallow/Hong Kong/D10-1161/2010	A	H5N1	2.3.2.1	1	HA1	Human	His	AGC13463.1
40160-V08B	A/barnswallow/HongKong/D10-1161/2010	A	H5N1	2.3.2.1	1	HA1+ HA2	Baculo-virus	His	AGC13463.1
40160-V08B1	A/barnswallow/HongKong/D10-1161/2010	A	H5N1	2.3.2.1	1	HA1+ HA2	Baculo-virus	His	AGC13463.1
11710-V08H1	A/Cambodia/R0405050/2007	A	H5N1	1.1	1	HA1	Human	His	ACI06178.1
11710-V08B	A/Cambodia/R0405050/2007	A	H5N1	1.1	1	HA1+ HA2	Baculo-virus	His	n.s.
11710-V08H	A/Cambodia/R0405050/2007	A	H5N1	1.1	1	HA1+ HA2	Human	His	ACI06178.1
40026-V08H1	A/Cambodia/S1211394/2008	A	H5N1	1.1	1	HA1	Human	His	ADM95445.1
40026-V08H	A/Cambodia/S1211394/2008	A	H5N1	1.1	1	HA1+ HA2	Human	His	ADM95445.1
11686-V08H1	A/chicken/Egypt/2253-1/2006	A	H5N1	2.2.1	1	HA1	Human	His	ABG81039.1
11712-V08H1	A/chicken/India/N	A	H5N1	2.2	1	HA1	Human	His	ABQ45850.1

	IV33487/06								
11712-V08B	A/chicken/India/NIV33487/06	A	H5N1	2.2	1	HA1+ HA2	Baculo-virus	His	ABQ45850.1
11712-V08H	A/chicken/India/NIV33487/06	A	H5N1	2.2	1	HA1+ HA2	Human	His	ABQ45850.1
40372-V08H1	A/chicken/Jilin/9/2004	A	H5N1	5 (1-2-5-6-8-9-like)	1	HA1	Human	His	n.s.
40372-V08B	A/chicken/Jilin/9/2004	A	H5N1	5 (1-2-5-6-8-9-like)	1	HA1+ HA2	Baculo-virus	His	AAT76166.1
40158-V08H1	A/chicken/VietNam/NCVD-016/2008	A	H5N1	7.1	1	HA1	Human	His	ACO07033.1
40158-V08B	A/chicken/VietNam/NCVD-016/2008	A	H5N1	7.1	1	HA1+ HA2	Baculo-virus	His	ACO07033.1
40158-V08B2	A/chicken/VietNam/NCVD-016/2008	A	H5N1	7.1	1	HA1+ HA2	Baculo-virus	His	ACO07033.1
40088-V08H1	A/chicken/Yamaguchi/7/2004	A	H5N1	2.5	1	HA1	Human	His	BAD89305.1
11700-V08H1	A/Common magpie/Hong Kong/2256/2006	A	H5N1	2.3.4	1	HA1	Human	His	ABJ96777.1
11700-V08H	A/Common magpie/Hong Kong/2256/2006	A	H5N1	2.3.4	1	HA1+ HA2	Human	His	ABJ96777.1
40044-V08H1	A/common magpie/Hong Kong/5052/2007	A	H5N1	2.3.2.1	1	HA1	Human	His	ACJ26242.1
40044-V08H	A/common magpie/Hong Kong/5052/2007	A	H5N1	2.3.2.1	1	HA1+ HA2	Human	His	ACJ26242.1
40001-V08H1	A/Duck/Hong Kong/p46/97	A	H5N1	0	1	HA1	Human	His	AAF02306.1
40001-V08H	A/Duck/Hong Kong/p46/97	A	H5N1	0	1	HA1+ HA2	Human	His	AAF02306.1
11698-V08H1	A/duck/Hunan/795/2002	A	H5N1	2.1.1	1	HA1	Human	His	ACA47835.1
11698-V08H	A/duck/Hunan/795/2002	A	H5N1	2.1.1	1	HA1+ HA2	Human	His	ACA47835.1
11701-V08H1	A/duck/Laos/3295/2006	A	H5N1	2.3.4	1	HA1	Human	His	ABG67978.1
11697-V08H1	A/Egypt/2321-NAMRU3/2007	A	H5N1	2.2.1	1	HA1	Human	His	ABP96850.1
11697-V08H	A/Egypt/2321-NAMRU3/2007	A	H5N1	2.2.1	1	HA1+ HA2	Human	His	ABP96850.1
40049-V08H1	A/Egypt/3300-NAMRU3/2008	A	H5N1	2.2.1.1	1	HA1	Human	His	ACI06185.1
11702-V08H1	A/Egypt/N05056/2009	A	H5N1	2.2.1	1	HA1	Human	His	ACT15357.1
11702-V08H	A/Egypt/N05056/2009	A	H5N1	2.2.1	1	HA1+ HA2	Human	His	ACT15357.1
40024-V08B	A/Goose/Guangdong/1/96	A	H5N1	0	1	HA1+ HA2	Baculo-virus	His	YP_308669.1
11690-V08H1	A/goose/Guiyang/337/2006	A	H5N1	4	1	HA1	Human	His	ABJ96698.1

11690-V08H	A/goose/Guiyang/337/2006	A	H5N1	4	1	HA1+HA2	Human	His	ABJ96698.1
11713-V08H1	A/Hong kong/213/2003	A	H5N1	1	1	HA1	Human	His	ABP51975.1
11713-V08H	A/Hong kong/213/2003	A	H5N1	1	1	HA1+HA2	Human	His	ABP51975.1
11689-V08H1	A/Hong Kong/483/97	A	H5N1	0	1	HA1	Human	His	AAC32099.1
11689-V08H	A/Hong Kong/483/97	A	H5N1	0	1	HA1+HA2	Human	His	AAC32099.1
40060-V08H1	A/Hubei/1/2010	A	H5N1	2.3.2.1	1	HA1	Human	His	AEO89181.1
40015-V08B	A/Hubei/1/2010	A	H5N1	2.3.2.1	1	HA1+HA2	Baculo-virus	His	AEO89181.1
40015-V08H	A/Hubei/1/2010	A	H5N1	2.3.2.1	1	HA1+HA2	Human	His	n.s.
40015-V08H1	A/Hubei/2011	A	H5N1	2.3.2.1	1	HA1	Human	His	n.s.
11060-V08H1	A/Indonesia/5/2005	A	H5N1	2.1.3.2	1	HA1+HA2	Human	His	ABW06108.1
11060-V08H2	A/Indonesia/5/2005	A	H5N1	2.1.3.2	1	HA1+HA2	Human	His	ABW06108.1
11694-V08H1	A/Japanese white-eye/Hong Kong/1038/2006	A	H5N1	2.3.4	1	HA1	Human	His	ABJ96775.1
11694-V08H	A/Japanese white-eye/Hong Kong/1038/2006	A	H5N1	2.3.4	1	HA1+HA2	Human	His	ABJ96775.1
40065-V08H1	A/Thailand/1(KAN-1)/2004	A	H5N1	1	1	HA1	Human	His	n.s.
40064-V07H	A/Thailand/1(KAN-1)/2004	A	H5N1	1	1	NA	Human	His	n.s.
40064-V07H-B	A/Thailand/1(KAN-1)/2004	A	H5N1	1	1	NA	Baculo-virus	His+Biot	n.s.
11061-V08H1	A/turkey/Turkey/1/2005	A	H5N1	2.2.1	1	HA1+HA2	Human	His	ABD73284.1
11061-V08H2	A/turkey/Turkey/1/2005	A	H5N1	2.2.1	1	HA1+HA2	Human	His	ABD73284.1
11062-V08H1	A/Vietnam/1194/2004	A	H5N1	1	1	HA1+HA2	Human	His	AAT73273.1
11062-V08H2	A/Vietnam/1194/2004	A	H5N1	1	1	HA1+HA2	Human	His	AAT73273.1
10003-V06H1	A/VietNam/1203/2004	A	H5N1	1	1	HA1	Human	His, Fc (mo)	AAW80717.1
10003-V06H3	A/VietNam/1203/2004	A	H5N1	1	1	HA1+HA2	Human	His+Fc (mo)	AAW80717.1
10003-V04H2	A/VietNam/1203/2004	A	H5N1	1	1	HA2	Human	Fc (mo)	AAW80717.1
40022-V08H1	A/Vietnam/UT31413II/2008	A	H5N1	2.3.4.3	1	HA1	Human	His	ADF83651.1
11709-V08H1	A/whooper swan/Mongolia/244/2005	A	H5N1	2.2	1	HA1	Human	His	ACZ36881.1
11709-V08H	A/whooper swan/Mongolia/244/2005	A	H5N1	2.2	1	HA1+HA2	Human	His	ACZ36881.1
40004-V08H1	A/Xinjiang/1/2006	A	H5N1	2.2	1	HA1	Human	His	ACJ68614.1

40004-V08H	A/Xinjiang/1/2006	A	H5N1	2.2	1	HA1+ HA2	Human	His	ACJ68614.1
pMG106	A/Cambodia/408 008/2005	A	H5N1	1	1	NS	Cell-free	His	n.s.
11699-V08H1	A/American green-winged teal/California/HK WF609/2007	A	H5N2	none	1	HA1	Human	His	ACF47563.1
11699-V08H	A/American green-winged teal/California/HK WF609/2007	A	H5N2	none	1	HA1+ HA2	Human	His	ACF47563.1
40014-V08H1	A/ostrich/South Africa/AI1091/200 6	A	H5N2	none	1	HA1	Human	His	ABQ24010.1
11696-V08H1	A/duck/Hokkaido/ 167/2007	A	H5N3	none	1	HA1	Human	His	BAG07130.2
11696-V08H	A/duck/Hokkaido/ 167/2007	A	H5N3	none	1	HA1+ HA2	Human	His	BAG07130.2
11717-V08H1	A/duck/NY/19125 5-59/2002	A	H5N8	none	1	HA1	Human	His	AAP72011.1
11717-V08H	A/duck/NY/19125 5-59/2002	A	H5N8	none	1	HA1+ HA2	Human	His	AAP72011.1
40164-V08H1	A/turkey/Ireland/1 378/1983	A	H5N8	none	1	HA1	Human	His	P11135
40164-V08B2	A/turkey/Ireland/1 378/1983	A	H5N8	none	1	HA2	Baculo-virus	His	ABI85117.1
40165-V08H1	A/chicken/Italy/22 A/1998	A	H5N9	none	1	HA1	Human	His	ABR37720.1
40165-V08B	A/chicken/Italy/22 A/1998	A	H5N9	none	1	HA1+ HA2	Baculo-virus	His	ABR37720.1
11723-V08H1	A/northern shoveler/Californi a/HKWF115/2007	A	H6N1		1	HA1	Human	His	ACE81692.1
11723-V08H	A/northern shoveler/Californi a/HKWF115/2007	A	H6N1		1	HA1+ HA2	Human	His	ACE81692.1
40166-V08H1	A/duck/Shantou/8 3/2000	A	H6N2		1	HA1	Human	His	ADG44842.1
pSN115	A/Turkey/Canada /1963	A	H6N2		1	NS	Cell-free	His	n.s.
pSN116	A/Turkey/Massac husetts/3740/196 5	A	H6N2		1	NS	Cell-free	His	n.s.
40027-V08B	A/chicken/Hong Kong/17/1977	A	H6N4		1	HA1+ HA2	Baculo-virus	His	CAC84244.1
40027-V08H1	A/chicken/Hong Kong/17/77	A	H6N4		1	HA1	Human	His	CAC84244.1
40027-V08H	A/chicken/HongK ong/17/77	A	H6N4		1	HA1+ HA2	Human	His	CAC84244.1
40167-V08H1	A/shearwater/Aus tralia/1/1973	A	H6N5		1	HA1	Human	His	ADD64203.1
40168-V08H1	A/mallard/Ohio/2 17/1998	A	H6N8		1	HA1	Human	His	ABO52049.1
40168-V08B	A/mallard/Ohio/2 17/1998	A	H6N8		1	HA1+ HA2	Baculo-virus	His	ABO52049.1
11722-V08H1	A/pintail	A	H8N4		1	HA1	Human	His	ABB87729.1

	duck/Alberta/114/1979								
11722-V08B	A/pintail duck/Alberta/114/1979	A	H8N4		1	HA1+HA2	Baculo-virus	His	ns.
11722-V08H	A/pintail duck/Alberta/114/1979	A	H8N4		1	HA1+HA2	Human	His	ABB87729.1
40036-V08H1	A/Chicken/Hong Kong/G9/97	A	H9N2		1	HA1	Human	His	AAF00701.1
40003-V08B	A/Duck/HongKong/448/78	A	H9N2		1	HA1+HA2	Baculo-virus	His	AAO46079.1
11719-V08H1	A/Guinea fowl/Hong Kong/WF10/99	A	H9N2		1	HA1	Human	His	AAO46082.1
11719-V08H	A/Guinea fowl/Hong Kong/WF10/99	A	H9N2		1	HA1+HA2	Human	His	AAO46082.1
11229-V08H	A/Hong Kong/1073/99	A	H9N2		1	HA1+HA2	Human	His	NP_859037.1
40174-V08H1	A/Hong Kong/35820/2009	A	H9N2		1	HA1	Human	His	n.s.
40174-V08B	A/Hong Kong/35820/2009	A	H9N2		1	HA1+HA2	Baculo-virus	His	ADC41853.1
11229-V08H1	A/HongKong/1073/99	A	H9N2		1	HA1	Human	His	NP_859037.1
40181-V08H1	A/shorebird/DE/261/2003	A	H9N5		1	HA1	Human	His	ABB87950.1
40181-V08B	A/shorebird/DE/261/2003	A	H9N5		1	HA1+HA2	Baculo-virus	His	ABB87950.1
11705-V08H1	A/duck/Yangzhou/906/2002	A	H11N2		1	HA1	Human	His	AAY85533.1
11705-V08H	A/duck/Yangzhou/906/2002	A	H11N2		1	HA1+HA2	Human	His	AAY85533.1
40188-V08H1	A/duck/England/1/1956	A	H11N6		1	HA1	Human	His	AGB50949.1
11704-V08H1	A/mallard/Alberta/294/1977	A	H11N9		1	HA1	Human	His	ABB87228.1
11704-V08H	A/mallard/Alberta/294/1977	A	H11N9		1	HA1+HA2	Human	His	ABB87228.1
40029-V08H1	A/mallard duck/Alberta/342/1983	A	H12N1		1	HA1	Human	His	ABB88099.1
40029-V08B	A/mallard duck/Alberta/342/1983	A	H12N1		1	HA1+HA2	Baculo-virus	His	ABB88099.1
40029-V08H	A/mallard duck/Alberta/342/1983	A	H12N1		1	HA1+HA2	Human	His	ABB88099.1
40189-V08H1	A/bar headed goose/Mongolia/143/2005	A	H12N3		1	HA1	Human	His	ACV86810.1
11718-V08H1	A/green-winged teal/ALB/199/1991	A	H12N5		1	HA1	Human	His	ABB88110.1
11718-V08H	A/green-winged teal/ALB/199/1991	A	H12N5		1	HA1+HA2	Human	His	ABB88110.1

11721-V08H1	A/black-headed gull/Netherlands/1/00	A	H13N8		1	HA1	Human	His	AAV91212.1
11721-V08B	A/black-headed gull/Netherlands/1/00	A	H13N8		1	HA1+ HA2	Baculo-virus	His	AAV91212.1
11721-V08H	A/black-headed gull/Netherlands/1/00	A	H13N8		1	HA1+ HA2	Human	His	AAV91212.1
11711-V08H1	A/black-headed gull/Sweden/5/99	A	H16N3		1	HA1	Human	His	AAV91217.1
11711-V08H	A/black-headed gull/Sweden/5/99	A	H16N3		1	HA1+ HA2	Human	His	AAV91217.1
40324-V08H1	A/flat-faced bat/Peru/033/2010	A	H18N11		1	HA1	Human	His	AGX84934.1
40324-V08B	A/flat-faced bat/Peru/033/2010	A	H18N11		1	HA1+ HA2	Baculo-virus	His	AGX84934.1
40140-V08H1	A/swine/Korea/PZ72-1/2006	A	H3N1		2	HA1	Human	His	ACS71642.1
40140-V08B	A/swine/Korea/PZ72-1/2006	A	H3N1		2	HA1+ HA2	Baculo-virus	His	ACS71642.1
11707-V08H1	A/Aichi/2/1968	A	H3N2		2	HA1	Human	His	AAA43178.1
11707-V08H	A/Aichi/2/1968	A	H3N2		2	HA1+ HA2	Human	His	AAA43178.1
40153-V08B	A/Babol/36/2005	A	H3N2		2	HA1+ HA2	Baculo-virus	His	n.s.
11056-V08H1	A/Brisbane/10/2007	A	H3N2		2	HA1	Human	His	ABW23353.1
11056-V08B	A/Brisbane/10/2007	A	H3N2		2	HA1+ HA2	Baculo-virus	His	ABW23353.1
11056-V08H	A/Brisbane/10/2007	A	H3N2		2	HA1+ HA2	Human	His	ABW23353.1
40118-V08H1	A/California/7/2004	A	H3N2		2	HA1	Human	His	ABW80975.1
40118-V08B	A/California/7/2004	A	H3N2		2	HA1+ HA2	Baculo-virus	His	ABW80975.1
40120-V08B	A/Fujian/411/2002	A	H3N2		2	HA1+ HA2	Baculo-virus	His	AFG72823.1
40152-V08B	A/Guangdong-Luohu/1256/2009	A	H3N2		2	HA1+ HA2	Baculo-virus	His	AFM72872.1
40116-V08H1	A/Hong Kong/1/1968	A	H3N2		2	HA1	Human	His	AAK51718.1
40116-V08B	A/Hong Kong/1/1968	A	H3N2		2	HA1+ HA2	Baculo-virus	His	Q91MA7
40146-V08B	A/Hong Kong/CUHK31987/2011	A	H3N2		2	HA1+ HA2	Baculo-virus	His	AGC13545.1
40101-V08H1	A/Memphis/1/68	A	H3N2		2	HA1	Human	His	ABB54514.1
40154-V08B	A/Moscow/10/1999	A	H3N2		2	HA1+ HA2	Baculo-virus	His	ABE73115.1
40043-V08H1	A/Perth/16/2009	A	H3N2		2	HA1	Human	His	ACS71642.1
40043-V08H	A/Perth/16/2009	A	H3N2		2	HA1+ HA2	Human	His	ACS71642.1
40058-V08H1	A/reassortant/IVR-155	A	H3N2		2	HA1	Human	His	ADI52838.1

40149-V08B	A/Sydney/5/1997	A	H3N2		2	HA1+ HA2	Baculo-virus	His	ACO95259.1
40354-V08H1	A/Texas/50/2012	A	H3N2		2	HA1	Human	His	AFH57070.1
40354-V08B	A/Texas/50/2012	A	H3N2		2	HA1+ HA2	Baculo-virus	His	EPI537015
40151-V08B	A/Victoria/208/2009	A	H3N2		2	HA1+ HA2	Baculo-virus	His	ADG21005.1
40058-V08B	A/Victoria/210/2009	A	H3N2		2	HA1+ HA2	Baculo-virus	His	ADI52838.1
40145-V08H1	A/Victoria/361/2011	A	H3N2		2	HA1	Human	His	AGB08328.1
11972-V08H1	A/Wisconsin/67/X-161/2005	A	H3N2		2	HA1	Human	His	ABO37609.1
11972-V08B	A/Wisconsin/67/X-161/2005	A	H3N2		2	HA1+ HA2	Baculo-virus	His	ACF41911.1
11972-V08H	A/Wisconsin/67/X-161/2005	A	H3N2		2	HA1+ HA2	Human	His	ABO37609.1
11715-V08H1	A/Wyoming/03/2003	A	H3N2		2	HA1	Human	His	ABX10525.1
11715-V08H	A/Wyoming/03/2003	A	H3N2		2	HA1+ HA2	Human	His	ABX10525.1
40059-V08H	A/X-31	A	H3N2		2	HA1+ HA2	Human	His	P03438
pSN108	A/Aichi/2/1968	A	H3N2		2	NS	Cell-free	His	n.s.
pSN112	A/New York/3087/2009	A	H3N2		2	NS	Cell-free	His	n.s.
pSN114	A/Swine/Minnesota/9088-2/1998(H3N2)	A	H3N2		2	NS	Cell-free	His	n.s.
40155-V08B	A/equine/Gansu/7/2008	A	H3N8		2	HA1	Baculo-virus	His	ACE81938.1
40155-V08H1	A/equine/Gansu/7/2008	A	H3N8		2	HA1	Human	His	ACE81938.1
pSN111	A/Equine/Miami/1/1963	A	H3N8		2	NS	Cell-free	His	n.s.
40008-V08H1	A/mallard duck/Alberta/299/1977	A	H4N4		2	HA1	Human	His	Q0A4G1
40008-V08B	A/mallard duck/Alberta/299/1977	A	H4N4		2	HA1+ HA2	Baculo-virus	His	ABB87495.1
40008-V08H	A/mallard duck/Alberta/299/1977	A	H4N4		2	HA1+ HA2	Human	His	Q0A4G1
11714-V08H1	A/mallard/Ohio/657/2002	A	H4N6		2	HA1	Human	His	ABI47995.1
11714-V08H	A/mallard/Ohio/657/2002	A	H4N6		2	HA1+ HA2	Human	His	ABI47995.1
11706-V08H1	A/Swine/Ontario/01911-1/99	A	H4N6		2	HA1	Human	His	AAG17429.1
11706-V08H	A/Swine/Ontario/01911-1/99	A	H4N6		2	HA1+ HA2	Human	His	AAG17429.1
pSN110	A/Duck/Czechoslovakia/1956	A	H4N6		2	NS	Cell-free	His	n.s.
40025-V08H1	A/chicken/Alabama/1/1975	A	H4N8		2	HA1	Human	His	P19695.1

40025-V08H	A/chicken/Alabama/1/1975	A	H4N8		2	HA1+ HA2	Human	His	P19695
40169-V08H1	A/turkey/Italy/4602/99	A	H7N1		2	HA1	Human	His	CAD38286.1
40170-V08H1	A/ruddy turnstone/New Jersey/563/2006	A	H7N2		2	HA1	Human	His	ACS68445.1
40170-V08B	A/ruddy turnstone/New Jersey/563/2006	A	H7N2		2	HA1+ HA2	Baculo-virus	His	ACS68445.1
40129-V08H1	A/chicken/SK/HR-00011/2007	A	H7N3		2	HA1	Human	His	ACA25329.1
40128-V08H1	A/turkey/Italy/214845/2002	A	H7N3		2	HA1	Human	His	CAF33017.1
40128-V08B	A/turkey/Italy/214845/2002	A	H7N3		2	HA1+ HA2	Baculo-virus	His	CAF33017.1
11212-V08H1	A/chicken/Netherlands/1/03	A	H7N7		2	HA1	Human	His	AAR02639.1
11212-V08B	A/chicken/Netherlands/1/03	A	H7N7		2	HA1+ HA2	Baculo-virus	His	AAR02639.1
40171-V08B	A/equine/Kentucky/1a/1975	A	H7N7		2	HA1+ HA2	Baculo-virus	His	ACL12085.1
11082-V08H1	A/Netherlands/219/03	A	H7N7		2	HA1	Human	His	AAR02640.1
11082-V08B	A/Netherlands/219/03	A	H7N7		2	HA1+ HA2	Baculo-virus	His	AAR02640.1
40172-V08H1	A/mallard/Netherlands/33/2006	A	H7N8		2	HA1	Human	His	EPI182113
40172-V08B	A/mallard/Netherlands/33/2006	A	H7N8		2	HA1+ HA2	Baculo-virus	His	ACR59554.1
pMG104	A/Beijing/1/2013	A	H7N9		2	NS	Cell-free	His	n.s.
40103-V08H1	A/Anhui/1/2013	A	H7N9		2	HA1	Human	His	AGJ51953.1
40103-V08H4	A/Anhui/1/2013	A	H7N9		2	HA1+ HA2	Human	His	EPI439507
40103-V08B	A/Anhui/1/2013	A	H7N9		2	HA1+ HA2	Baculo-virus	His	EPI439507
40103-V08H	A/Anhui/1/2013	A	H7N9		2	HA1+ HA2	Human	His	EPI439507
40105-V08H1	A/Hangzhou/1/2013	A	H7N9		2	HA1	Human	His	AGI60301.1
40105-V08B	A/Hangzhou/1/2013	A	H7N9		2	HA1+ HA2	Baculo-virus	His	AGI60301.1
40105-V08H	A/Hangzhou/1/2013	A	H7N9		2	HA1+ HA2	Human	His	AGI60301.1
40123-V08B	A/Hangzhou/3/2013	A	H7N9		2	HA1+ HA2	Baculo-virus	His	EPI442713
40106-V08B1	A/Pigeon/Shanghai/S1069/2013	A	H7N9		2	HA1	Baculo-virus	His	n.s.
40106-V08H1	A/Pigeon/Shanghai/S1069/2013	A	H7N9		2	HA1	Human	His	n.s.
40106-V08H1	A/Pigeon/Shanghai/S1069/2013	A	H7N9		2	HA1	Human	His	n.s.
40106-V08B	A/Pigeon/Shanghai/S1069/2013	A	H7N9		2	HA1+ HA2	Baculo-virus	His	n.s.
40106-V08H	A/Pigeon/Shanghai/S1069/2013	A	H7N9		2	HA1+ HA2	Human	His	n.s.

40109-V07H	A/Shanghai/1/2013	A	H7N9		2	NA	Human	His	n.s.
40109-VNAHC	A/Shanghai/1/2013	A	H7N9		2	NA	Human	n.s.	n.s.
40104-V08B1	A/Shanghai/1/2013	A	H7N9		2	HA1	Baculo-virus	His	n.s.
40104-V08H1	A/Shanghai/1/2013	A	H7N9		2	HA1	Human	His	n.s.
40104-V08B	A/Shanghai/1/2013	A	H7N9		2	HA1+HA2	Baculo-virus	His	n.s.
40104-V08H	A/Shanghai/1/2013	A	H7N9		2	HA1+HA2	Human	His	n.s.
40104-V08H4	A/Shanghai/1/2013	A	H7N9		2	HA1+HA2	Human	His	n.s.
40111-V08B	A/Shanghai/2/2013	A	H7N9		2	NP	Baculo-virus	His	AGL44439.1
40239-V08B	A/Shanghai/2/2013	A	H7N9		2	HA1+HA2	Baculo-virus	His	n.s.
40239-V08H	A/Shanghai/2/2013	A	H7N9		2	HA1+HA2	Human	His	n.s.
40126-V08B	A/Shanghai/4664T/2013	A	H7N9		2	HA1+HA2	Baculo-virus	His	AGI60292.1
40125-V08B	A/Zhejiang/1/2013	A	H7N9		2	HA1+HA2	Baculo-virus	His	EPI443034
40325-V08B	A/Zhejiang/DTID-ZJU10/2013	A	H7N9		2	HA1+HA2	Baculo-virus	His	AHA11500.1
40325-V08H	A/Zhejiang/DTID-ZJU10/2013	A	H7N9		2	HA1+HA2	Human	His	AHA11500.1
11693-V08H1	A/duck/Hong Kong/786/1979	A	H10N3		2	HA1	Human	His	BAF46762.1
11693-V08B	A/duck/Hong Kong/786/1979	A	H10N3		2	HA1+HA2	Baculo-virus	His	BAF46762.1
11693-V08H	A/duck/Hong Kong/786/1979	A	H10N3		2	HA1+HA2	Human	His	BAF46762.1
40360-V08H1	A/duck/Hunan/S11205/2012	A	H10N3		2	HA1	Human	His	AGO87051.1
40184-V08H1	A/mallard/Minnesota/Sg-00194/2007	A	H10N3		2	HA1	Human	His	ACT84107.1
40184-V08B	A/mallard/Minnesota/Sg-00194/2007	A	H10N3		2	HA1+HA2	Baculo-virus	His	ACT84107.1
pMG105	A/Chicken/Germany/n/1949	A	H10N7		2	NS	Cell-free	His	n.s.
40351-V08H1	A/duck/Guangdong/E1/2012	A	H10N8		2	HA1	Human	His	n.s.
40359-V08B	A/Jiangxi-Donghu/346/2013	A	H10N8		2	HA1+HA2	Baculo-virus	His	EPI497477
40359-VNAB	A/Jiangxi-Donghu/346/2013	A	H10N8		2	HA1+HA2 (HA0)	Baculo-virus	n.s.	EPI497477
40028-V08H1	A/duck/Hong Kong/562/1979	A	H10N9		2	HA1	Human	His	ABI84469.1
40028-V08B	A/duck/Hong Kong/562/1979	A	H10N9		2	HA1+HA2	Baculo-virus	His	ABI84469.1
40028-V08H	A/duck/Hong Kong/562/1979	A	H10N9		2	HA1+HA2	Human	His	ABI84469.1
40192-V08H1	A/mallard/Astrakh	A	H14N5		2	HA1	Human	His	P26136

	an/263/1982								
40193-V08H1	A/Australian shelduck/Western Australia/1756/1983	A	H15N2		2	HA1	Human	His	ABB90704.1
40193-V08B	A/Australian shelduck/Western Australia/1756/1983	A	H15N2		2	HA1+ HA2	Baculo-virus	His	ABB90704.1
11720-V08H1	A/duck/AUS/341/1983	A	H15N8		2	HA1	Human	His	ABB88132.1
11720-V08H	A/duck/AUS/341/1983	A	H15N8		2	HA1+ HA2	Human	His	ABB88132.1
11053-V01H2	B/Florida/4/2006	B	FluB			HA2	Human	Fc (hu)	ACA33493.1
11053-V04H2	B/Florida/4/2006	B	FluB			HA2	Human	Fc (mo)	ACA33493.1
11053-V08H	B/Florida/4/2006	B	FluB			HA1+ HA2	Human	His	ACA33493.1
11053-V08H1	B/Florida/4/2006	B	FluB			HA1	Human	His	ACA33493.1
11716-V08H	B/Malaysia/2506/2004	B	FluB			HA1+ HA2	Human	His	ACO05957.1
11716-V08H1	B/Malaysia/2506/2004	B	FluB			HA1	Human	His	ACO05957.1
40016-V08H	B/Brisbane/60/2008	B	FluB			HA1+ HA2	Human	His	ACN29380.1
40016-V08H1	B/Brisbane/60/2008	B	FluB			HA1	Human	His	ACN29383.1
40157-V08H1	B/Yamagata/16/1988	B	FluB			HA1	Human	His	n.s.

a HA1, hemagglutinin head; HA2, hemagglutinin stem; NA, neuraminidase; NP, nucleoprotein; NS, non-structural protein

b mo, mouse; hu, human; biot, biotin; n.s., not supplied by vendor

c n.s., not supplied by vendor
